# Decoupled evolution of floral traits and climatic preferences in a clade of Neotropical Gesneriaceae

**DOI:** 10.1186/s12862-015-0527-6

**Published:** 2015-11-10

**Authors:** Martha Liliana Serrano-Serrano, Mathieu Perret, Maïté Guignard, Alain Chautems, Daniele Silvestro, Nicolas Salamin

**Affiliations:** Department of Ecology and Evolution, University of Lausanne, 1015 Lausanne, Switzerland; Swiss Institute of Bioinformatics, Quartier Sorge, 1015 Lausanne, Switzerland; Conservatoire et Jardin botaniques de la Ville de Genève and Laboratory of Plant Systematics and Biodiversity, University of Geneva, Chemin de l’Impératrice, 1, 1292 Chambésy Geneva, Switzerland; Department of Plant and Environmental Sciences, University of Gothenburg, Carl Skottsbergs gata 22B, 413 19 Gothenburg, Sweden

**Keywords:** Brazilian Atlantic forest, Hummingbird pollination, Traitgram, Resupination, Pollination syndrome, Trait evolution, Comparative methods

## Abstract

**Background:**

Major factors influencing the phenotypic diversity of a lineage can be recognized by characterizing the extent and mode of trait evolution between related species. Here, we compared the evolutionary dynamics of traits associated with floral morphology and climatic preferences in a clade composed of the genera *Codonanthopsis, Codonanthe* and *Nematanthus* (Gesneriaceae). To test the mode and specific components that lead to phenotypic diversity in this group, we performed a Bayesian phylogenetic analysis of combined nuclear and plastid DNA sequences and modeled the evolution of quantitative traits related to flower shape and size and to climatic preferences. We propose an alternative approach to display graphically the complex dynamics of trait evolution along a phylogenetic tree using a wide range of evolutionary scenarios.

**Results:**

Our results demonstrated heterogeneous trait evolution. Floral shapes displaced into separate regimes selected by the different pollinator types (hummingbirds versus insects), while floral size underwent a clade-specific evolution. Rates of evolution were higher for the clade that is hummingbird pollinated and experienced flower resupination, compared with species pollinated by bees, suggesting a relevant role of plant-pollinator interactions in lowland rainforest. The evolution of temperature preferences is best explained by a model with distinct selective regimes between the Brazilian Atlantic Forest and the other biomes, whereas differentiation along the precipitation axis was characterized by higher rates, compared with temperature, and no regime or clade-specific patterns.

**Conclusions:**

Our study shows different selective regimes and clade-specific patterns in the evolution of morphological and climatic components during the diversification of Neotropical species. Our new graphical visualization tool allows the representation of trait trajectories under parameter-rich models, thus contributing to a better understanding of complex evolutionary dynamics.

**Electronic supplementary material:**

The online version of this article (doi:10.1186/s12862-015-0527-6) contains supplementary material, which is available to authorized users.

## Background

Throughout the evolutionary process, lineages may experience divergent modifications of their phenotype and genome that culminate with the establishment of separate species. Modeling the evolution of species traits can help to elucidate the likely sequence of diversification events that lead to phenotypically diverse groups of species [1]. Traits related to different niche axes are expected to follow different evolutionary trajectories that may reflect different selection pressures, genetic constraints or stages of diversification determining the order in which the different ecological axes are partitioned during species divergence [2]. For example, patterns of trait divergence during the diversification of live oaks (*Ceanothus*) in California suggested that traits related to local scale coexistence show an early divergence in the group, while traits related to large scale habitat display a later or throughout differentiation [3]. Although theoretical work supports similar scenarios [1] empirical support for this model in other plant groups and across different types of traits still needs to be evaluated.

Hypotheses about the ordering of trait divergence during the evolution of a lineage can be complemented by investigating the mode and tempo of trait diversification within lineages [4]. For instance, initially rapid morphological evolution followed by relative stasis [5] could be the result of new ecological opportunities accompanied by density-dependent slowdowns in species diversification [6, 7]. To explore this process, trait evolution can be reconstructed along the branches of phylogenetic trees to detect heterogeneity in evolutionary rates through time, across lineages or in relation to discrete characters [8–10]. Furthermore, Ornstein-Uhlenbeck (OU) models can be used to describe bounded phenotypic evolution, where single or multiple selective regimes pull phenotypes towards optimum values [11]. In plants, these models have helped to understand the evolutionary dynamics of flower morphology [12] and climatic niche [13]. Multiple studies have identified heterogeneous rates of evolution across climate dimensions in specific clades [14] and, at a larger scale, rates of niche evolution within major groups of angiosperms that are dependent on the type of growth form [15]. The possibility of testing multiple models to reveal complex patterns of trait evolution during species diversification is an important advantage to understand the dynamics of trait evolution and differential evolution among traits [7, 16, 17]. However, the fit between the current models and the real evolutionary processes is widely discussed [5, 18], and the power for selecting models depends on the number of taxa, the shape of the phylogeny, and the presence of measurements errors [19].

In this study, we investigate the evolutionary history of floral morphology and climatic preferences in a clade of epiphytic plants belonging to the genera *Codonanthopsis, Codonanthe,* and *Nematanthus* (hereafter referred to as the CCN clade) of the Gesneriaceae family. This group provides an excellent opportunity to compare patterns of evolutionary diversification between these niche axes. CCN clade exhibits a remarkable floral diversity in shape, size and orientation reflecting potential adaptation to different pollinators including bees and various hummingbirds [20–25]. Furthermore, CCN clade is widely distributed throughout most Neotropical rainforest but present the highest species richness and level of range overlap in the Brazilian Atlantic Forest (BAF) [26, 27]. Understanding how these morphological or climatic axes of niche differentiation have evolved in this plant group could shed light on the way speciation processes are building Neotropical biodiversity. First, we test if traits related to flower shape and size better fits a pollinator shift model involving transitions between adaptive peaks defined by pollinator morphology and behavior [12], or if flowers have diversified regardless of the pollinator type. Second, we determine if the evolution of climatic preferences is best explained by a model with distinct ecological optima [13] or a model with more labile evolution of climatic preferences among closely related species [14]. To address these questions we first infer phylogenetic relationships among the species using multi-gene DNA sequences. We quantify the floral morphology and climatic space occupied by the group and, examine the tempo and mode of evolution of different traits in the CCN group using current models of trait evolution. We finally develop a new approach to visualize the estimated trait evolution by proposing an alternative way to incorporate information from complex models. Our results suggest that phenotypic evolution of this group is described by a variety of processes with different mode, time and lineage-specific effects. A new visualization of complex models of trait evolution further allows a better understanding of the particular processes at play in this group of Neotropical plants.

## Methods

### Phylogenetic analyses

Taxonomic sampling included 46 out of the 52 species in the group, as well as 13 outgroup species. Six molecular makers, two nuclear (ITS and *ncpGS*) and four plastid regions (*atpB-rbcL* spacer*, rpl16* intron*, rps16* intron*, trnL-trnF* spacer) were sequenced and aligned for a final DNA matrix of 4.484 bp. We reconstructed phylogenetic relationships and relative divergence times using MrBayes and BEAST [28, 29]. Best fitting nucleotide substitution models were estimated with the *phymltest* function in the R package ape [30]. Log-normal uncorrelated relaxed clock and the Yule speciation priors were set for the analyses. We used a maximum clade credibility (MCC) tree and a sample of high posterior probability trees from the BEAST results for later analyses. Finally, we examined the evolution of three binary traits (geographic distribution, pollination syndromes, and floral orientation) by reconstructing their ancestral states in the R package corHMM [31]. Detailed description of the molecular dataset, phylogenetic reconstructions, and ancestral state estimation are provided in the Additional file 1: Appendix S1.

### Morphometric data

Thirteen quantitative traits representing different aspects of the floral shape and size (Additional file 1: Figure S1) were measured for 38 species out of the 46 included in the phylogenetic analysis (Additional file 1: Table S3). Measurements were obtained from 2 to 15 flowers (average = 5) collected from wild individuals or cultivated plants at the Botanical Garden of Geneva (Switzerland). Collection permits were granted by the CNPq in Brazil (CMC 038/03) and the ANAM in Panama (SC/P-43-10). Floral material was not available for *Codonanthopsis dissimulata,* five species of *Codonanthe* and two *Nematanthus* (*C. crassifolia, C. calcarata, C. gibbosa, C. erubescens, C. luteola, N. kautskyi,* and *N. lanceolatus).* However, original descriptions of the species and photographic material available at Mauro Peixoto website (www.brazilplants.com) and at the Gesneriaceae Image Library (http://gesneriads.ua.edu/image-library/) indicates that the missing species do not represent exceptional morpho-types of the group. Thus, we can postulate that our quantitative measurements are representative of the morphological diversity of each clade and that we do not miss important variation because of the species lacking morphological data. Species positions in morphological space was quantified with a principal component analysis (PCA) using the R package *Ade4* [32] based on the covariance matrix of mean values for each species. We used non-transformed data, but log-transformed PCA patterns were also examined and led to a very similar morphospace (see Additional file 1: Figure S5).

### Species climatic preferences

Climatic parameters for each species of the CCN clade were estimated from occurrence data and layers for climatic data. Locality descriptions were derived from the labels on specimens examined in more than 50 herbaria (Additional file 1: Table S4). Georeference coordinates were generated for all localities that could be attributed to a precise geographic entity. We completed the dataset for species occurring outside Brazil with additional georeferenced specimens retrieved from GBIF (data.gbif.org, 2012-02-06). Occurrence data for *C. corniculata*, *C. elegans* and *N. serpens* were not included in the analysis due to limited number of herbarium material and uncertainty on their native distribution. A total of 2,240 occurrence points remained after manual checking and removal of duplicated points with a median of 15 occurrences per species. Climatic data (elevation and 19 bioclimatic variables) were extracted directly from Bioclim environmental layers [33] on a 30 arc-second resolution grid (~1 km^2^ at the equator). Occurrence data and their associated climatic information extracted from the Bioclim layers can be found at http://www2.unil.ch/phylo/files/serranoetal15_bioclim.xls. We used these climatic parameters to represent the species distribution along climatic gradients. We denoted this climatic space as the species preferences, although these preferences may be limited by interactions with other species, historical factors or dispersal limitation [34]. To explore the relative position of each species in the climatic space of the CCN clade, we performed a PCA using outlying mean index ordination (OMI) [35], which assigns a mean position of each species on the climatic space, as implemented in *Ade4* package in R [32]. Values for each of the 19 Bioclim variables, plus altitude, were used for the ordination.

### Models of continuous trait evolution

We examined the patterns of trait evolution by using the MCC tree and multiple models, which span from a single Brownian motion rate of evolution (BM with single σ^2^), BM with variable rate through time (decreasing or increasing σ^2^) including early burst (EB) to Ornstein-Uhlenbeck models. All these models were fitted using the *fitContinuous* function in geiger R package [36]. The Ornstein-Uhlenbeck models were fitted using either a single selective regime (one single σ^2^, selection strength α and optimum parameter θ) or multi-regime processes. For the latter case, we tested four different OU models using the *OUwie* function in the OUwie package [11] (Additional file 1: Table S6 for acronyms), each with two different regime categories: pollination syndromes and geographic distribution. These categories were treated as binary characters, following the reconstructions described in Additional file 1: Appendix S1. Delta-AIC (ΔAIC) and Akaike weights (ω) were calculated for model comparisons. Furthermore, Blomberg’s K [37] was estimated as a measure of phylogenetic signal using the R package phytools [38]. The outgroup species were pruned from the trees in all the morphology and climatic preferences analyses.

In addition to the models of trait evolution, a multi-rate BM model was tested using the R package geiger to identify rate changes among lineages [36]. This flexible method aims to identify changes on rates of continuous trait evolution among lineages. The analyses were performed on 100 phylogenetic trees randomly sampled from the BEAST posterior distribution and the MCMC was run for 100,000 generations, sampling every 100 generations and excluding the first 25 % for burn-in. Each run provides posterior distributions of branch-wise rate estimates and probabilities of rate shifts. We compared the fit of the multi-rates model against the fit of alternative BM and OU models by comparing their AIC value. The multi-rates BM analysis used reversible jump MCMC (rjMCMC) and we estimated the AIC values for each model in two ways. First, we directly took the best likelihood value sampled over the rjMCMC samples and calculate the corresponding AIC value based on the number of parameters of the model. Second, we mapped the set of branches at each rate category with the *make.era.map* function, and used the non-censored approach implemented in the *brownie.lite* function (phytools R package) [38] to produce the maximum likelihood estimate and calculate the AIC values. A bias towards parameter-rich models can occur during the model selection process if measurement error is not considered [19]. We performed additional model comparisons to test for such effects by estimating the amount of measurement error present in our dataset. Such estimation is not possible for multi-rates BM model but we used the values estimated under single BM model estimation for these cases.

### Visualization of continuous trait evolution

The parameters estimated under the best fitting model of trait evolution describe the process numerically, but a graphical visualization of trait evolutionary trajectories remains difficult to picture. Recent graphical methods (often referred to as “traitgrams”) help to visualize phenotypic evolution by plotting a phylogenetic tree against trait values [39]. However, such methods are limited to the BM model, partly due to the difficulty of inferring ancestral states under more complex evolutionary models. Here, we implemented an alternative approach to display the dynamics of trait evolution along a phylogenetic tree. We achieved this by forward-time simulations under complex models of trait evolution and analytical interpolation between ancestral states. The purpose of these reconstructions is to provide a graphical representation of the expected continuous evolution of phenotypes given a phylogeny and a set of parameters describing the evolutionary process. For each trait, we recorded the topological placement and magnitude of parameter changes across the tree, e.g. rates, selection strength and optima, depending on the model (Table 1). We then simulated 100 realizations of trait evolution under the optimized model and parameter values along the BEAST MCC tree (see Appendix S2 for more details). All forward simulations started by sampling a random number from a normal distribution with parameters estimated from a posterior distribution of root states obtained with the *fitContinuousMCMC* function in geiger [36]. Other parameters (σ^2^, α, θ) were taken from the best fitting model.

**Table 1:** Results of model fitting for the morphological and climate PC axes

Models	AICc	ΔAIC	ω
Floral size – Morphology PC1			
Brownian Motion	190.9718	38.7133	0.0000
Ornstein-Uhlenbeck	187.7321	35.4736	0.0000
OU alternative^a^	172.9199	20.6614	0.0000
Early Burst (DC)	193.3461	41.0876	0.0000
Early burst (AC)	187.7321	35.4736	0.0000
Multiple rates	152.2585	0.0000	1.0000
Floral shape – Morphology PC2			
Brownian Motion	104.3669	24.8268	0.0000
Ornstein-Uhlenbeck	106.6641	27.1240	0.0000
OU alternative^a^	79.5401	0.0000	0.9996
Early Burst (DC)	106.7412	27.2011	0.0000
Early burst (AC)	106.6641	27.1240	0.0000
Multiple rates	95.2293	15.6892	0.0004
Mean and seasonality in temperature - Climate PC1			
Brownian Motion	134.5776	10.1545	0.0059
Ornstein-Uhlenbeck	135.6864	11.2633	0.0034
OU alternative^a^	124.4231	0.0000	0.9465
Early Burst (DC)	136.9015	12.4784	0.0018
Early burst (AC)	136.5797	12.1566	0.0022
Multiple rates	130.7428	6.3197	0.0402
Precipitation seasonality - Climate PC2			
Brownian Motion	115.0848	19.9517	0.0000
Ornstein-Uhlenbeck	100.5829	5.4498	0.0548
OU alternative^a^	100.5768	5.4437	0.0549
Early Burst (DC)	117.4086	22.2755	0.0000
Early burst (AC)	100.5829	5.4498	0.0548
Multiple rates	95.1331	0.0000	0.8355

^a^OU alternative corresponds to Ornstein–Uhlenbeck models with different numbers of parameters. Here, only the model with the best AIC value is reported, see the full set of OU models in summarized in the Additional file 1: Table S6. (AICc = corrected Akaike Information Criterion values, ΔAIC Delta AICc and ω = Akaike weights)

We used the simulated trait values at the internal nodes and at the tips to plot traitgrams displaying the reconstructed trait evolution. In their standard implementation traitgrams connects the trait values between nodes by a straight line to draw the edges of a tree [39, 40], consistently with the expected anagenetic trait evolution under a constant rate BM model. However, under more complex models such as OU processes, the expected trait value *x*(*t*) at a time *t* between two nodes of age *t*_*i*_ < *t < t*_*j*_ and trait values *x*(*t*_*i*_) and *x*(*t*_*j*_) respectively, deviates from a straight line. This deviation occurs because the evolutionary trajectory of the trait under an OU model depends not only on the evolutionary rate, but also on the strength of selection and the relative distance from the optimum [41]. The expected anagenetic evolution of a trait under an OU model is described by the Ornstein-Uhlenbeck bridge (personal communication) [42] and can be obtained for any time *t* as:

$$ x(t)=\theta +\frac{\left(x\left({t}_i\right)-\theta \right) \sinh\;\left(\alpha \left({t}_j-t\right)\right)}{ \sinh\;\left(\alpha \left({t}_j-{t}_i\right)\right)}+\frac{\left(x\left({t}_i\right)-\theta \right) \sinh\;\left(\alpha \left(t-{t}_i\right)\right)}{ \sinh\;\left(\alpha \left({t}_j-{t}_i\right)\right)},\kern0.5em \mathrm{f}\mathrm{o}\mathrm{r}\kern1em {t}_i<t<{t}_j $$

where θ is the optimum and α the strength of selection. The parameters θ and α can vary across the edges of a tree if the model includes different selective regimes.

We generated multiple realizations of the trait evolution under the different BM and OU models and parameter settings estimated for the four traits. These were combined to plot the 95 % CI of the trait ranges through time (i.e., the minimum and maximum trait values across clades at any time *t*). These plots thus provide a graphical representation of how the range of potential trait values are expected to change through time, given a fixed tree topology and a complex model of trait evolution. Single realizations of the simulated process, resembling the conventional traitgrams, facilitate the understanding of regime- or clade-specific patterns. The method is implemented in an R script available at http://www2.unil.ch/phylo/files/software/plot_traitgram_serranoetal15.R.

## Results

### Phylogenetic analyses

**Fig. 1:**
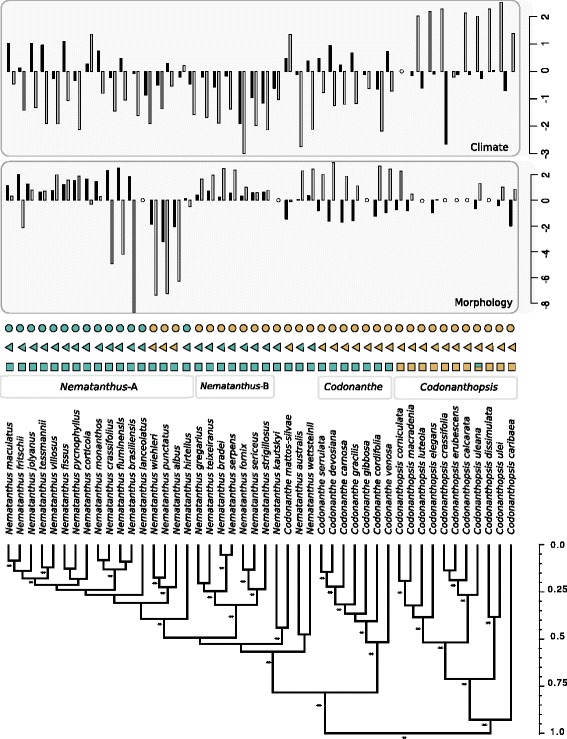
Maximum clade credibility tree for CCN ingroup species. Asterisk on branches are Bayesian posterior probabilities >0.99. Scale on the bottom right is relative time. Subclades are indicated by gray-shaded boxes. Binary traits are indicated for each species on top of the tree. Geographical distribution: green square = BAF, yellow square = other biomes. Pollination syndromes: green triangle = hummingbird, yellow triangle = bee pollinated, and floral orientation: green circle = resupinate, yellow circle = non-resupinate. Principal component values for morphology and climate are enclosed in gray frame, upper panels. Gray and black vertical bars represent PC1 and PC2, respectively

The tree topologies reconstructed from MrBayes and BEAST were congruent and generally highly supported (see Additional file 1: Figures S2 and S6). The BEAST analysis (Fig. 1, see Additional file 1: Figure S2 for tree with outgroups) indicated that the initial divergence in the CCN lineage occurred between the *Codonanthopsis* clade and the remaining taxa that are all endemic to the BAF. The *Codonanthopsis* clade is widely distributed in Central America, the Caribbean, the Andes and the Amazonian basin, but does not occur in the BAF except for the widespread *C. uleana*, which extends its range into the northern part of this biome. The BAF clade is composed of the two sister clades corresponding to the *Codonanthe s.s.* (seven species) and *Nematanthus* (27 species). Within *Nematanthus*, the sister species *N. australis* and *N. wettsteinii* first diverged from the remaining *Nematanthus,* which includes two sister clades identified as *Nematanthus A* (17 species) and *Nematanthus B* (7 species). High posterior probabilities supported these clades (1.0), while ambiguous placements were observed for *C. mattos-silvae, N. kaustkyi* and *N. hirtellus* species. Ancestral reconstructions for geography, pollination syndrome and floral orientation using the preferred model (ER) showed few state changes, mainly involving complete clade transitions (Additional file 1: Figure S3).

### Floral morphology and climatic preferences space

The first and second axes of the PCA on the floral traits explained 85.1 % of the variance (PC1 70.7 %; PC2 14.4 %, Additional file 1: Figure S4a and Table S5). PC1 mainly reflected variation in flower size with loadings of same sign and approximately equal value for all measurements. Variation in flower size (PC1) was particularly extensive in the *Nematanthus*-A clade (Additional file 1: Figure S4a). PC2 had a positive loading for stamen, pistil and tube lengths, vertical diameter of corolla tube, and a negative loading for the diameters of limb and corolla opening and restriction before nectary chamber. PC2 therefore mainly represented variation in flower shape, with a positive value indicating tubular and narrowly opened corolla, while a negative value indicates more campanulate corolla with inserted stamen and broad limb and opening. All species with positive value of PC2 belong to the genus *Nematanthus.* Their flowers are pigmented in red, orange or yellow and match well the definition of the syndrome of hummingbird pollination (Fenster et al. 2004), as confirmed by field studies for 10 species (see Additional file 1: Table S2). Species with negative PC2 values belong to the *Codonanthe, Codonanthopsis* and to a specific subclade within *Nematanthus*-A*.* Their flowers have several features traditionally associated with bee pollination such as the creamy corolla, brownish dots inside the tube (nectary guide), inserted stamen and developed inferior lobes forming a landing platform for insects. However, to our knowledge, no field studies have confirmed bee pollination for these species.

The first two PCs for climatic variables accounted for 69.1 % of the variance (PC1 54.30 %, PC2 14.84 %, Additional file 1: Figure S4b). PC1 reflected mainly the variation in temperature (Bio9, Bio11, Bio4 and Bio7) with positive values indicating warmer mean temperatures with low seasonal variation and negative values showing strong variability in temperature through the year. PC2 was mainly correlated with precipitation (Bio15 and Bio14). Positive PC2 values indicated high seasonal variability in precipitation regimes, while negative values showed high precipitation on the wettest month and low seasonality (see Additional file 1: Figure S4b for species climatic values). Climatic space indicated a clear separation between the *Codonanthopsis* species and the BAF lineages (*Codonanthe* plus *Nematanthus* clades), in agreement with their distinct geographical distribution.

### Models of continuous trait evolution

The models of trait evolution indicated that distinct evolutionary processes have influenced trait divergence in the CCN group. Individual axes of floral morphology (size and shape) and climatic preferences (temperature and precipitation) variation seemed to have evolved independently. The incorporation of measurement error in our model comparisons did not lead to any bias towards parameter-rich models and the four phenotypic axes present no changes in the preferred models when accounting for it (Additional file 1: Table S7).

**Fig. 2:**
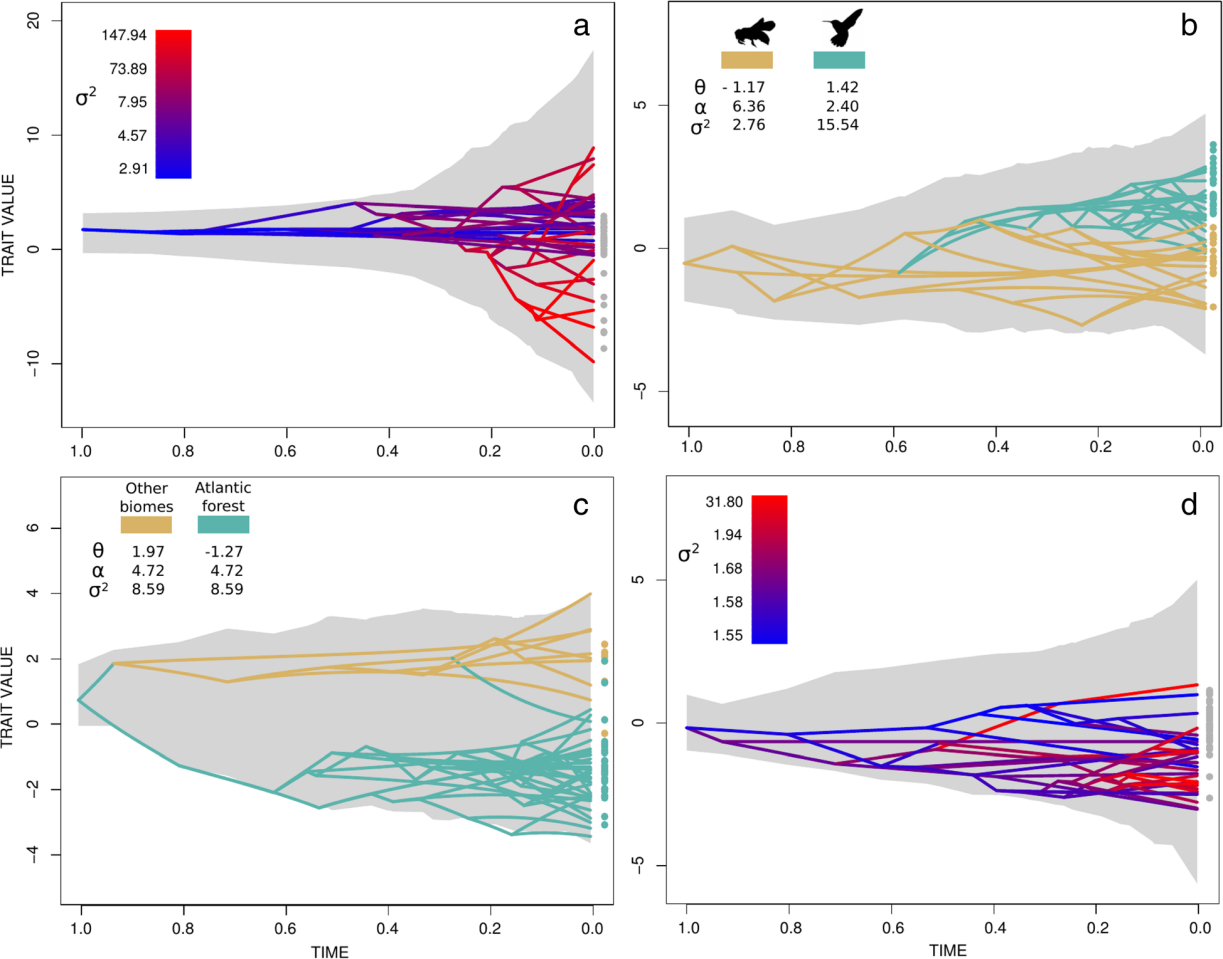
Simulated trait space and traitgrams (under specific models in Table 1) for morphological and climatic traits. The Y-axis corresponds to the trait values for the species, and should not be confound with the variances between them, thus the gray-shaded area is the 95 % CI of simulated trait ranges. Panel (**a**), floral size (morphological PC1) with multiple Brownian motion model. Panel (**b**), floral shape (morphological PC2) with regimes of the OUMVA model defined as bee and hummingbird pollinated species. Panel (**c**), mean and seasonality in temperature (climatic PC1) with regimes in a OUM model defined as Atlantic forest and other biomes. Panel (**d**), precipitation seasonality (climatic PC2) with multiple Brownian motion model. Colored scale in (**a**) and (**d**) correspond to branch-specific rates of trait evolution. Colors in (**b**) and (**c**) correspond to multiple regimes. Parameters θ, α and σ^2^ correspond to the optimum, strength of selection and rates of evolution, respectively according to the model specification. Points at the right of each panel indicates the observed trait values in all species analyzed

**Fig. 3:**
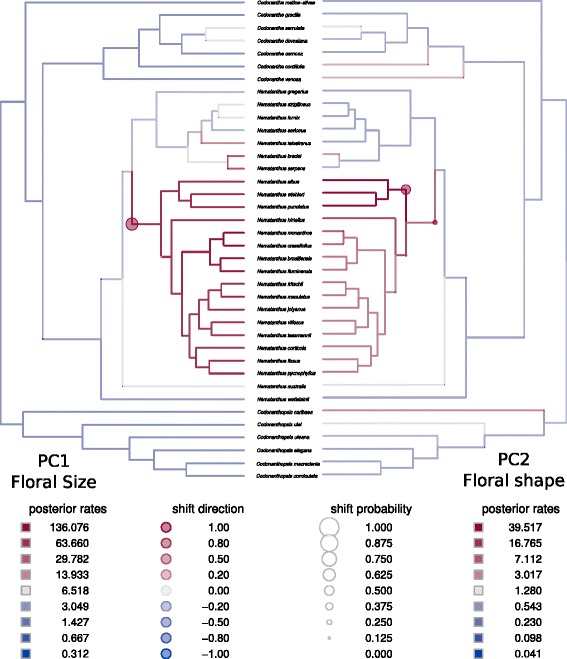
Posterior comparisons of rate of morphological trait evolution in the CCN group. Hue and size of circles at branches denote posterior support for a rate shift at the indicated branch. Larger and redder circles suggest higher posterior support for an upturn in evolutionary rate (see Eastman et al. 2011)⁠. Branches in the phylogeny are colored such that rates not deviant from the median are shaded gray; rates below (or above) the median are shaded blue (or red). Rates corresponding to each hue are indicated in the legend, as well as shift probabilities and directions

The evolution of floral size (PC1) was best described by the multi-rates BM model with branch-specific rates (Table 1). The ΔAIC is very large suggesting that the alternative models poorly represent the evolution of the floral size. The posterior probabilities for the rates of evolution across the phylogeny supported one rate shift in this trait (Fig. 3), which is associated with the origin of the *Nematanthus-*A clade (shift probability > 0.625). This shift involved a strong rate increase and represents a deviation from a constant BM process, which is consistent with the observed low values of Blomberg's K statistic for PC1 (mean of 0.496 with 25 % and 75 % quantiles of 0.463 and 0.546, respectively). According to the parameter estimated by the best fitting model, the visualization of floral size evolution shows narrow trait ranges in the early stages of the diversification of the CCN group, followed by a large increase in trait ranges due to the single clade shift (Fig. 2a). From the simulated trait trajectories, and because the best model was based on BM, the increase in trait range can be symmetric (positive or negative axis). However, the empirical data suggested that only deviation towards bigger floral sizes occurred (negative loadings). The evolution of the floral shape (PC2) was best explained by an OUMVA model with two regimes, which are defined by the pollination types. Each regime had different rates of evolution (σ^2^), optima (θ) and strength of selection (α). Bee-pollinated species (negative loadings in the PC2) evolved in a constrained way, with a narrower dispersion from the optimum value, whereas hummingbird-pollinated species (positive loadings in PC2) explored a wider trait space during their evolution (Fig. 2b). Phylogenetic reconstruction indicated a single origin of hummingbird syndrome at the root of the *Nematanthus* clade and more recent reversal to bee syndrome in a clade of three species (*N. albus, N. punctatus*, and *N. wiehleri,* Additional file 1: Figure S3). Floral shape constraint agrees with the high Blomberg's K values obtained for the morphological PC2 (mean value of 1.323 with 25 % and 75 % quantiles of 1.122 and 1.547, respectively).

**Fig. 4:**
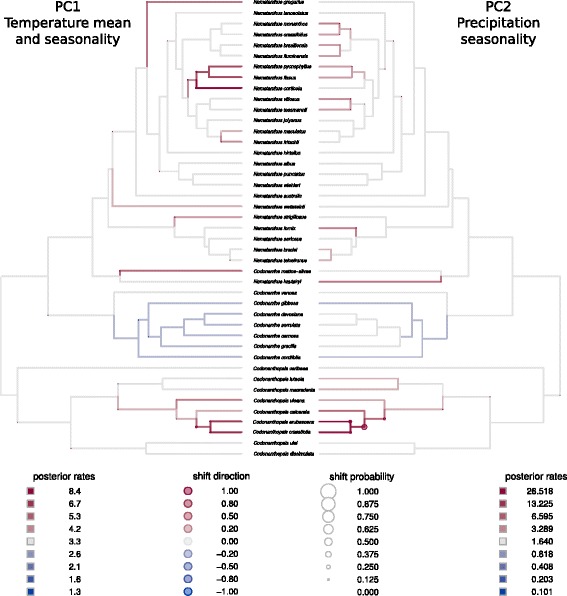
Posterior comparisons of rate of climatic preferences evolution in the CCN group. See caption of Fig. 3

The evolution of climatic preferences also showed different dynamics among its components. The best model for temperature (climate PC1) was OUM, with different optima between the two geographic distributions (i.e., Central, northern South America and Amazonian basin versus Brazilian Atlantic forest), but equal rates of evolution and selection coefficient per regime. The trait space for temperature displayed a strongly bounded evolution with a slow rate of change (Fig. 2c). The extent of change in positive and negative loadings appeared to be symmetrical. Estimates of Blomberg's K statistic for climatic PC1 indicated a mean value of 0.477 with 25 % and 75 % quantiles of 0.376 and 0.541, respectively. Multi-rates BM model was the best fitted for precipitation seasonality (climatic PC2), but posterior evidence for a rate shift was weak. Only a minor increase in rates of climatic differentiation between the sister species *C. erubescens* and *C. crassifolia* was detected (see Fig. 4). The evolution of trait space for the climatic PC2 appeared as a constant increase of phenotypic space over time (Fig. 2d). This was consistent with the results in Fig. 4, showing that large shifts in the trait values are rare. Estimates of Blomberg's K values for climatic PC2 ranged from 0.357 and 0.459 (25 % and 75 % quantiles respectively) with a mean value of 0.408.

## Discussion

Testing the order and extent of trait divergence during the evolution of a clade helps to understand the relative importance of separate morphological and climatic trajectories, as well as the possible drivers of species diversification. We combine a phylogenetic analysis and multiple models of trait evolution with simulations under the selected models, in order to comprehensively understand these different trajectories of trait evolution in the CCN group. Our results suggest that phenotypic evolution of this group is described by a variety of processes with different mode, time and lineage-specific effects. A new visualization of complex models of trait evolution further allow a better understanding of the particular processes at play in this group of Neotropical plants.

### Floral evolution dynamics

The inference of evolutionary models and estimation of plausible trait ranges for floral morphology revealed contrasting patterns during the evolutionary history of the CCN group. Floral size, represented by morphological PC1, has evolved in a complex fashion. The estimated trait range through time showed an initial period of narrow divergence, followed by a marked increase in trait ranges associated with the accelerated evolution of flower size within the clade *Nematanthus*-A (Fig. 2a). We did not detect evidence of a slow down in the rate of evolution of the PC1, showing that divergence in floral size continues throughout the diversification of the *Nematanthus-A* lineage during the Miocene (24 Mya, 95 % HPD 33.45 – 9.30 Mya [27]). This result contrasts with the classical model of adaptive radiation, where morphological evolution is initially rapid and then slows through time [5]. Our analyses were not aimed at investigating whether the CCN clade is a case of adaptive radiation, however the lack of a slow down in the evolutionary trajectory of floral size suggest that morphological space is not yet filled.

The increase in rates of evolution of floral size detected at the base of the *Nematanthus*-A clade coincides with the evolution of floral resupination (see the placement of the rate shift in Fig. 3, and the most probable transition to resupinate flowers occurring in almost all species of the clade except for *N. albus, N. wiehleri* and *N. punctatus* in Fig. S3). A direct consequence of the evolution of flower resupination is a change of pollen placement on the body of the pollinator. In resupinate species belonging to the *Nematanthus-*A clade, pollen is primarily transported on different parts of the ventral side of hummingbirds [20, 23]. In contrast, non-resupinate *Nematanthus* place most of their pollen on the top of the bill [22]. Therefore, flower resupination and the associated shift of pollen deposition could have stimulated the diversification of floral size in *Nematanthus*-A clade by creating new opportunities for species coexistence while sharing pollinators as was shown in another community of hummingbird-pollinated plants [43]. Extending this analysis to other Gesneriaceae clades such as *Glossoloma* and *Crantzia* that independently evolved resupinate flowers pollinated by hummingbirds, would provide a mean to further test the positive effect of resupination on flower diversification [44, 45]. An additional feature of the *Nematanthus*-A clade is its contrasting altitudinal distribution compared with *Nematanthus*-B (median values of 524 m and 957 m, respectively). In the BAF, these two altitudinal levels have contrasting hummingbird assemblages, with lowland communities presenting a greater richness of hummingbirds and a corresponding higher heterogeneity in bill size (short-billed trochilines and long-billed hermits) compared with the highland sites [21, 22]. Indeed, *Nematanthus*-A species in lowland rainforest are visited by both Trochilinae (non-hermit) and long-billed hermit hummingbirds (*Phaethornis* and *Ramphodon*), whereas species from *Nematanthus-B* mainly rely on Trochilinae hummingbirds for pollination (see Additional file 1: Table S2). We suggest that the interaction of *Nematanthus-A* species with broader range of hummingbird types and bill lengths in the lowland forest could also have provided more opportunities for flower size diversification in this clade.

Contrary to flower size, the evolution of the morphological PC2, floral shape, was preferentially supported by OU models that contained differential strength of selection, optima and rate parameters between hummingbird and bee pollination syndromes. The evolution of floral shape, from funnel-shaped corolla with expanded lobes in *Codonanthopsis* and *Codonanthe* clades to narrow-mouth and pouched corollas in most *Nematanthus* species could have been differentially constrained and maintained by functional groups of pollinators in agreement with the pollination syndrome concept [46]. Hummingbird-pollinated species showed a lower strength of selection and higher rates of trait change than species with a bee pollination syndrome (parameters in Fig. 2b), suggesting that hummingbirds might interact with a wider range of flower shapes than bees. The visualization of the floral shape trait space in this group provides a convenient tool that helps to better understand the dynamics of the OU process and the evolution of the independent optima.

Overall, our results suggest that the evolutionary trajectory of floral morphology in the CCN group may be constrained in shape (PC2), with possible evolutionary transitions from one functional group of pollinators to another following the pollinator shift model [12]. The transitions between these two phenotypic clusters may reflect selection to improve the interaction with better pollinators [46], and/or to avoid less efficient floral-pollinator associations [47, 48]. In comparison, variation in floral size (PC1) could be more related to character displacement and the establishment of mechanical isolation between co-occurring species sharing a same functional group of pollinators [49, 50]. This model of flower evolution based on competition for hummingbird pollination has been shown to generate phenotypic overdispersion within communities of Andean Solanaceae [51]. Testing this prediction in the CCN clade would require to further investigate whether co-occurring species are more different in flower size than expected due to chance across different sites in the BAF.

### Climatic evolution dynamics

The evolution of climatic preferences, represented in PC1 by temperature, is best explained by an OU model (Table 1). The different optima in the OUM model indicate the differentiation between BAF and other rainforests in the Neotropical region. However, models accounting for different rates of evolution and/or strength of selection are not preferred. This result potentially indicates that species preferences in temperature evolve at a similar pace, and that the strength of selection is comparable between groups inhabiting different regions. This pattern could reflect specific regional variation and dispersal limitation among the CCN lineages. The graphical representation of the evolution of the temperature preference showed an early differentiation between the selective regimes with rare subsequent transitions from one biome to the other (Fig. 2c). In contrast, the multi-rate BM model best explained the precipitation preferences, which are represented by PC2. The estimated rates for this component were higher than the overall rates in PC1 (see parameters in Fig. 2c and branch-specific estimations in Fig. 4), suggesting that species preferences for precipitation seasonality might change more rapidly than temperature preferences which are mainly biome specific.

Several evolutionary studies have reported pronounced ecological niche differentiation, concentrated in particular lineages [13, 14, 52] or associated with distinct species traits [53, 54], which suggest an important role of climatic changes during speciation. Our results for climatic differentiation suggest an early biome separation of the CCN clades (BAF and the rest of the Neotropics), followed by a divergence along different local conditions of precipitation seasonality. Although the role of this climatic component in speciation would need to be further investigated, this result is consistent with previous studies showing that floristic turnover in the Atlantic forests is largely correlated with distance from the ocean and rainfall distribution patterns [55, 56] and that allopatric speciation could have been particularly frequent along this climatic gradient [57, 58].

## Conclusions

Our investigation of the mode and tempo of trait evolution in the CCN clade provided evidence for a contrasting relevance of morphological and ecological divergences during species diversification. Two traits – flower shape and temperature preferences – were segregated into adaptive zones associated with different functional group of pollinators or biogeographic regions. First, floral shape evolution was constrained reflecting the selection to different functional groups of pollinators (i.e. hummingbirds vs insects). Second, divergence in temperature was linked with the colonization of the BAF biome at an early stage of the evolution of the CCN group. On the contrary, two other trait components – flower size and precipitation preferences – evolved at a higher rate, with no recent slowdown. Changes in floral size occurred mainly in a specific subclade including species with resupinate flowers and lowland distribution, whereas evolutionary changes of precipitation seasonality likely took place tree-wide and throughout the entire CCN diversification. The contrasting patterns between the constrained evolution of floral shape (i.e. pollination syndrome) and the diversification of precipitation seasonality across time agree with the habitat first rule model proposed by Ackerly et al. (2006) suggesting early divergence of traits that allow species to co-occur (alpha niche) and a throughout diversification of traits defining macrohabitats (beta niche). We found, however, that both flower morphology and climatic preferences diversified along different axes that are better fitted by distinct models of evolution. Our new implementation for visualization allowed us to graphically represent trait evolutionary histories, using models that go beyond the simple BM and can potentially better capture complex evolutionary dynamics. This approach represents an enhancement of current methods to plot the phenotypic space through time, with the simulation part being of potential use as a predictive tool for measuring the adequacy of alternative models. Finally, our study calls for a broader phylogenetic scale analysis to unravel the mechanisms driving such evolutionary processes and their potential effect on the remarkable species richness in the Neotropics.

## Additional file

Additional file 1:
**Appendix S1.** Phylogenetic analysis and relative divergence time estimation. **Appendix S2.** Visualization of continuous trait evolution. **Table S1.** List of species sampled, voucher information, Genbank accession numbers and binary trait information. **Table S2.** Pollinator, locality and source of information for *Nematanthus* species. **Table S3.** Mean values for floral traits. **Table S4.** Index Herbariorum acronyms and names. **Table S5.** PC loadings for morphological and climatic preferences. **Table S6.** AICc values for the complete set of OU models. **Table S7.** Model fit comparison correcting for measurement error (ME). **Figure S1.** Floral measurements. **Figure S2.** Phylogenetic reconstruction with outgroup species. **Figure S3.** Ancestral state reconstructions. **Figure S4.** Principal component analysis for floral traits (a) and climatic preferences (b). **Figure S5.** Comparison of phenotypic space and multi-rates BM model from raw and log-transformed measurements. **Figure S6.** Credibility tree from MrBayes with outgroup sequences included. (PDF 4115 kb)
