# Supplementary material for: Decoupled evolution of floral traits and climatic preferences in a clade of Neotropical Gesneriaceae
Source: BMC Evol Biol. 2015 Nov 10;15:247. doi: 10.1186/s12862-015-0527-6 (PMC4641406; doi:10.1186/s12862-015-0527-6)
Supplement: Additional file 1: — Appendix S1. Phylogenetic analysis and relative divergence time estimation. Appendix S2. Visualization of continuous trait evolution. Table S1. List of species sampled, voucher information, Genbank accession numbers and binary trait information. Table S2. Pollinator, locality and source of information for Nematanthus species. Table S3. Mean values for floral traits. Table S4. Index Herbariorum acronyms and names. Table S5. PC loadings for morphological and climatic preferences. Table S6. AICc values for the complete set of OU models. Table S7. Model fit comparison correcting for measurement error (ME). Figure S1. Floral measurements. Figure S2. Phylogenetic reconstruction with outgroup species. Figure S3. Ancestral state reconstructions. Figure S4. Principal component analysis for floral traits (a) and climatic preferences (b). Figure S5. Comparison of phenotypic space and multi-rates BM model from raw and log-transformed measurements. Figure S6. Credibility tree from MrBayes with outgroup sequences included. (PDF 4115 kb) [file 12862_2015_527_MOESM1_ESM.pdf]

SUPPLEMENTARY INFORMATION

Decoupled evolution of floral traits and climatic preferences in a clade of neotropical Gesneriaceae.

Serrano-Serrano ML, Perret M, Guignard M, Chautems A, Silvestro D, and Salamin N.

Contents

Appendix S1. Phylogenetic analysis and relative divergence time estimation 2
Appendix S2. Visualization of continuous trait evolution 3

Table S1. List of species sampled, voucher information, Genbank accession numbers and binary trait information. 5
Table S2. Pollinator, locality and source of information for *Nematanthus* species........ 6
Table S3. Mean values for floral traits 8
Table S4. Index Herbariorum acronyms and names 9
Table S5. PC loadings for morphological and climatic preferences 10
Table S6. AICc values for the complete set of OU models 11
Table S7. Model fit comparison correcting for measurement error (ME) 12

Figure S1. Floral measurements 13
Figure S2. Phylogenetic reconstruction with outgroup species 14
Figure S3. Ancestral state reconstructions 15
Figure S4. Principal component analysis for floral traits (a) and climatic preferences (b) 17
Figure S5. Comparison of phenotypic space and multi-rates BM model from raw and log-transformed measurements 18
Figure S6. Credibility tree from MrBayes with outgroup sequences included 19
References 20

Appendix S1. Phylogenetic analysis and relative divergence time estimation

The *Codonanthopsis, Codonanthe* and *Nematanthus* clade has ca. 52 species and is distributed throughout the Neotropical rainforest with a center of diversity in the Brazilian Atlantic forest (BAF). Recent phylogenetic and biogeographic analyses of the Neotropical Gesneriaceae have shown that the CCN group is divided in two sister clades (Perret et al. 2013). One is composed of *Codonanthe* sensu stricto and *Nematanthus* (39 species) that are endemic to the BAF (Chautems 2009), while the other includes *Codonanthopsis* (13 species) that occurs throughout the tropical rainforest of Central America, northern South America and Amazonia (Chautems and Perret 2013). We sampled 27 *Nematanthus,* 8 *Codonanthe* and 11 *Codonanthopsis* species, as well as 13 outgroup species representing different Episceae genera and other related Neotropical tribes such as Gesnerieae (*Gesneria humilis*), Gloxineae (*Kohleria spicata*) and Sinningieae (*Sinningia schiffneri*). This taxonomic sample includes all known species in the three genera CCN except *C. anisophylla, C. chiricana, N. mirabilis, N. striatus,* and the probable hybrid species *N. kuhlmannii* and *N. mattosianus* for which plant material was not available. All sampled species were sequenced for two nuclear (ITS, *ncpGS*) and four plastid regions (*atpB-rbcL* spacer, *rpl16* intron, *rps16* intron, *trnL-trnF* spacer) using the procedure described in Perret et al. (2013). Voucher information and Genbank numbers for *trnL-trnF* and *rps16* sequences are published in Perret et al. (2013). Genbank numbers for other markers are provided in Table S1. We used MAFFT (Katoh and Kuma 2002) and Guidance (Penn et al. 2010) to align sequences and subsequently remove all sites with a confidence score below 0.8; the final matrix contained 4,484 bp. We identified the GTR+G model of substitution as the best model for each DNA region, using AIC method as implemented in the *phymltest* function in R (*ape* package; Paradis et al. 2004). The only exception was the *ncpGS* gene, which was modeled with HKY85+G.

We used these models in Bayesian inference (BI: MrBayes 3.2; Ronquist et al. 2012) and all model parameters were unlinked across gene partitions. We performed two runs of BI for the complete alignment matrix, where each run consisted of four chains of 10^7 generations, sampling every 10^3 steps. We determined chain convergence and burn-in length (20% of the sampled generations) by examining trace plots of each parameter in Tracer v.1.4 (Rambaut and Drummond 2007). A consensus tree was calculated by removing the burn-in period

and combining the two runs. Phylogenetic relationships and relative divergence times were estimated with a relaxed clock model applying uncorrelated log-normal prior distribution for the rates of substitution and a Yule prior on the rates of speciation using BEAST (Drummond et al. 2012). Each DNA region was treated as a separate data partition, allowing parameters of each region to be unlinked. Three independent analyses were performed by including either all 46 species sampled, the 38 species with morphological data or the 43 species with climatic data (see below). All three analyses included two independent runs of $2*10^7$ generations each. The CCN clade was constrained to be monophyletic based on previous studies (Clark et al. 2012; Perret et al. 2013). Due to the absence of closely related fossils in the CCN clade, difficulties to align sequences from fast evolving genes in higher taxa, and the multiple issues of secondary calibrations (Sauquet et al. 2012), an accurate absolute divergence-time estimation for this group is problematic. However, our analyses did not require absolute times of divergence because our goal is to compare the evolution of different traits within the CCN clade. We therefore used BEAST to produce ultrametric trees by setting the prior for the time of the most recent common ancestor of CCN clade to an almost fixed root of 1 (normal distribution with mean=1, sd=1e-06). We also carried out a MrBayes (v3.2; Ronquist et al. 2012) analysis for comparison (See Figure S6). Finally, we examined the evolution of three binary traits: geographic distribution, pollination syndromes, and floral orientation (see Table S1). The biogeographic data was obtained from Perret et al. (2013), while information on pollination syndromes and resupination was obtained from available field observations of pollinators (see Table S2) and observations on living plants. Ancestral state reconstructions were performed using the Maximum Clade Credibility (MCC) tree and the function *rayDISC* (root=maddfitz) in the corHMM R package (Beaulieu et al. 2013).

Appendix S2. Visualization of continuous trait evolution

Trait simulations for each trait used the MCC tree of the BEAST analyses used the functions *sim.rates* (phytools R package, Revell 2012), for the multiple BM model, and *OUwie.sim* (Ouwie R package, Beaulieu et al. 2012) for the OU models. The *sim.rates* function allows for multiple BM rates of evolution, here estimated by rjMCMC (geiger R package, see above) and mapped on each branch of the phylogenetic tree. The *OUwie.sim* function requires OU parameters and tree painted with the defined selective regimes. All parameters were set according to the model selected for each trait (Table 1) and the root states were based on BM maximum likelihood estimation, even for OU models, because of the impossibility to estimate root state under these models.

Table S1. List of species sampled, voucher information,and floral orientation (FO, 0= non-resupinated, 1= resupinated). Genbank accession numbers in italics correspond to sequences submitted with this manuscript, non-italics correspond to sequences reported in Perret et al. 2013. Binary trait information for geographic distribution (GD, 0= other biomes, 1= BAF), pollination syndrome (PS, 0= bee-pollinated and 1= hummingbird-pollinated).

Taxon	Voucher	Origin	trnLF	rps16	atpB-rbcL	ITS	ncpGS	rpl16	GD	PS	FO
Lesia savannarum (C.V. Morton) J.L. Clark & J.F. Sm.	R. Keller 4029 (G)	NA	*KT958424*	*KT958428*	*NA*	*KT958313*	*KT958258*	NA	NA	NA	NA
Chrysothemis pulchella (Donn exSims) Decne.	A.O.Araujo & al. 602 (G)	Cultivated CJBG AC-2305 (Ecuador)	GQ383544	GQ383582	*KT958447*	*KT958331*	*KT958276*	*KT958388*	NA	NA	NA
Codonanthe calcarata(Miq.) Hanst.	NA	Cultivated CJBG AC-3305 (Northern South America)	JX195726	JX195810	*KT958432*	*KT958314*	*KT958259*	*KT958371*	0	0	0
Codonanthe caribaea Urb.	A. Chautems & M. Perret 99-042 (G)	Cultivated CJBG 19b (Caribbean region)	JX195727	JX195811	*NA*	*KT958315*	*KT958260*	*KT958372*	0	0	0
Codonanthe carnosa (Gardner) Hanst.	A. Chautems & M. Perret 08-001 (G)	Cultivated CJBG AC-2703 (Brazil)	JX195728	JX195812	*KT958433*	*KT958316*	*KT958261*	*KT958373*	1	0	0
Codonanthe cordifolia Chautems	A. Chautems & M. Perret 10-103 (G)	Cultivated CJBG AC-1201 (Brazil)	JX195730	JX195814	*KT958434*	*KT958317*	*KT958262*	*KT958374*	1	0	0
Codonanthe corniculata Wiehler	A. Chautems & M. Perret 99-043 (G)	Cultivated CJBG AC-2607 (Peru)	JX195731	JX195815	*KT958435*	*KT958318*	*KT958263*	*KT958375*	0	0	0
Codonanthe crassifolia (H.Focke) C.V.Morton	A. Chautems & M. Perret 06-102 (G)	CultivatedMP-1010 (Neotropics)	JX195732	NA	*KT958436*	*KT958319*	*KT958264*	*KT958376*	0	0	0
Codonanthe devosiana Lem.	A. Chautems & M. Perret 99-010 (G)	Cultivated CJBG AC-1147 (Brazil)	JX195734	JX195817	*KT958437*	*KT958320*	*KT958265*	*KT958377*	1	0	0
Codonanthe elegans Wiehler	A. Chautems & M. Perret 07-101 (G)	Cultivated CJBG AC-2511 (Belize)	JX195735	JX195818	*NA*	*KT958321*	*KT958266*	*KT958378*	0	0	0
Codonanthe erubescens Wiehler	NA	Cultivated CJBG AC-3320 (Ecuador)	JX195736	JX195819	*KT958438*	*KT958322*	*KT958267*	*KT958379*	0	0	0
Codonanthe gibbosa J. Rossini & Chautems	L. Kollmann & al. 4784 (MBML)	Brazil	JX195737	JX195820	*KT958439*	*KT958323*	*KT958268*	*KT958380*	1	0	0
Codonanthe gracilis (Mart.) Hanst.	A. Chautems & M. Perret 99-003 (G)	Cultivated CJBG AC-1139 (Brazil)	JX195740	JX195823	*KT958440*	*KT958324*	*KT958269*	*KT958381*	1	0	0
Codonanthe luteola Wiehler	NA	Cultivated CJBG AC-3505 (Panama)	JX195741	JX195824	*KT958441*	*KT958325*	*KT958270*	*KT958382*	0	0	0
Codonanthe macradenia Donn.Sm.	A. Chautems & M. Perret 10-107 (G)	Cultivated CJBG 10a (Central America & Colombia)	JX195742	JX195825	*KT958442*	*KT958326*	*KT958271*	*KT958383*	0	0	0
Codonanthe mattos-silvae Chautems	A. Chautems & M. Perret 99-033 (G)	Cultivated CJBG AC-2001 (Brazil)	JX195743	JX195826	*KT958443*	*KT958327*	*KT958272*	*KT958384*	1	0	0
Codonanthe serrulata Chautems	A. Chautems & M. Perret 99-045 (G)	Cultivated CJBGAC-1307 (Brazil)	AJ439826	GQ383583	*KT958444*	*KT958328*	*KT958273*	*KT958385*	1	0	0
Codonanthe uleana Fritsch	A. Chautems & M. Perret 02-105 (G)	Cultivated CJBG AC-1308 (Brazil)	JX195744	NA	*KT958445*	*KT958329*	*KT958274*	*KT958386*	1	0	0
Codonanthe venosa Chautems	A. Chautems & M. Perret 99-006 (G)	Cultivated CJBG AC-1701 (Brazil)	JX195745	JX195827	*KT958446*	*KT958330*	*KT958275*	*KT958387*	1	0	0
Codonanthopsis dissimulata (H.E.Moore) Wiehler	M. Perret & A. Chautems 79 (G)	Cultivated MP-1089 (Brazil)	JX195753	JX195835	*KT958451*	*KT958335*	*KT958280*	*NA*	0	0	0
Codonanthopsis ulei Mansf.	A. Chautems & M. Perret 07-002 (G)	Cultivated CJBG AC-2801 (Brazil)	JX195754	JX195836	*KT958452*	*KT958336*	*KT958281*	*KT958392*	0	0	0
Columnea orientandina (Wiehler) L.P. Kvist& L.E. Skog	A. Chautems & M. Perret 09-102 (G)	Cultivated CJBG 22d (Ecuador & Peru)	JX195748	JX195830	*KT958448*	*KT958332*	*KT958277*	*KT958389*	NA	NA	NA
Columnea ulei Mansf.	A. Chautems & M. Perret 10-109 (G)	Cultivated CJBG AC-2803 (Brazil)	JX195750	JX195832	*KT958449*	*KT958333*	*KT958278*	*KT958390*	NA	NA	NA
Corytoplectus capitatus (Hook.)Wiehler	NA	CultivatedCJBG s.n. (Venezuela)	JX195752	JX195834	*KT958450*	*KT958334*	*KT958279*	*KT958391*	NA	NA	NA
Drymonia serrulata (Jacq.)Mart.	A.O.Araujo & al. 601 (G)	Cultivated CJBG AC-1311 (Brazil)	GQ383548	GQ383587	*KT958453*	*KT958337*	*KT958282*	*KT958393*	NA	NA	NA
Episcia cupreata (Hook.) Hanst.	A. Chautems & M. Perret 01-026 (G)	Cultivated CJBG AC-2509 "Bolivia copper" (Bolivia)	AJ439824	JX195841	*KT958454*	*KT958338*	*KT958283*	*KT958394*	NA	NA	NA
Gesneria humilis L.	A. Chautems & M. Perret 97-020 (G)	CultivatedCJBG s.n. (Cuba & Haiti)	AJ439821	GQ383588	*KT958455*	*KT958339*	*KT958284*	*KT958395*	NA	NA	NA
Kohleria spicata (Kunth) Oerst.	A. Chautems & M. Perret 97-021 (G)	CultivatedCJBG s.n. (Neotropics)	AJ439820	GQ383594	*KT958456*	*KT958340*	*KT958285*	*KT958396*	NA	NA	NA
Nautilocalyx melittifolius (L.) Wiehler	A. Chautems & M. Perret 01-025 (G)	CultivatedCJBG s.n. (Caribbean region)	AJ439829	GQ383606	*KT958481*	*KT958368*	*KT958310*	*KT958421*	NA	NA	NA
Nematanthus albus Chautems	A. Chautems & M. Perret 99-012 (G)	Cultivated CJBG AC-1369 (Brazil)	JX195765	JX195848	*KT958457*	*KT958341*	*KT958286*	*KT958397*	1	0	0
Nematanthus australis Chautems	A. Chautems & M. Perret 99-011 (G)	Cultivated CJBG AC-1146 (Brazil)	JX195766	JX195849	*KT958458*	*KT958342*	*KT958287*	*KT958398*	1	1	0
Nematanthus bradei	Chautems & Perret 99-030 (G)	Cultivated MP-270 (Brazil)	*KT958425*	*KT958429*	*NA*	*KT958343*	*NA*	*NA*	1	1	0
Nematanthus brasiliensis (Vell.) Chautems	A. Chautems & M. Perret 07-303 (G)	Cultivated CJBG AC-2802 (Brazil)	JX195767	JX195850	*KT958459*	*KT958344*	*KT958288*	*KT958399*	1	1	1
Nematanthus corticola Schrad.	A. Chautems & M. Perret 99-013 (G)	Cultivated CJBG AC-1323 (Brazil)	JX195768	JX195851	*KT958460*	*KT958345*	*KT958289*	*KT958400*	1	1	1
Nematanthus crassifolius (Schott) Wiehler	A. Chautems & M. Perret 07-403 (G)	Cultivated CJBG AC-1355 (Brazil)	JX195769	JX195852	*KT958461*	*KT958346*	*KT958290*	*KT958401*	1	1	1
Nematanthus fissus	Chautems & Perret 99-029 (G)	Cultivated MP-68 (Brazil)	*KT958426*	*KT958430*	*NA*	*KT958347*	*KT958291*	*NA*	1	1	1
Nematanthus fluminensis(Vell.) Fritsch	A. Chautems & M. Perret 99-020 (G)	Cultivated CJBG AC-1167 (Brazil)	JX195770	JX195853	*KT958462*	*KT958348*	*NA*	*KT958402*	1	1	1
Nematanthus fornix (Vell.) Chautems	A. Chautems & M. Perret 10-101 (G)	Cultivated CJBG AC-1157 (Brazil)	JX195771	JX195854	*KT958463*	*KT958349*	*KT958292*	*KT958403*	1	1	0
Nematanthus fritschii Hoehne	A. Chautems & M. Perret 07-404 (G)	Cultivated CJBG AC-1105 (Brazil)	JX195772	JX195855	*KT958464*	*KT958350*	*KT958293*	*KT958404*	1	1	1
Nematanthus gregarius D.L.Denham	A. Chautems & M. Perret 99-019 (G)	Cultivated CJBG AC-1116 (Brazil)	JX195773	JX195856	*KT958465*	*KT958351*	*KT958294*	*KT958405*	1	1	0
Nematanthus hirtellus (Schott)Wiehler	NA	Cultivated MP-331 (Brazil)	JX195774	JX195857	*KT958466*	*KT958352*	*KT958295*	*KT958406*	1	1	1
Nematanthus jolyanus (Handro) Chautems	A. Chautems & M. Perret 99-028 (G)	Cultivated MP-85 (Brazil)	JX195775	JX195858	*KT958467*	*KT958353*	*KT958296*	*KT958407*	1	1	1
Nematanthus kautskyi Chautems & J. Rossini	A. Chautems & M. Perret 99-040 (G)	Cultivated MP-434 (Brazil)	JX195776	JX195859	*KT958468*	*KT958354*	*KT958297*	*KT958408*	1	1	0
Nematanthus lanceolatus	NA	Cultivated CJBG AC-2002 (Brazil)	*KT958427*	*KT958431*	*NA*	*KT958355*	*KT958298*	*NA*	1	1	1
Nematanthus maculatus (Fritsch) Wiehler	A. Chautems & M. Perret 07-405 (G)	Cultivated CJBG AC-1125 (Brazil)	JX195777	JX195860	*KT958469*	*KT958356*	*NA*	*KT958409*	1	1	1
Nematanthus monanthos (Vell.) Chautems	A. Chautems & M. Perret 08-601 (G)	Cultivated CJBG AC-1216 (Brazil)	JX195778	JX195861	*KT958470*	*KT958357*	*KT958299*	*KT958410*	1	1	1
Nematanthus punctatus Chautems	A. Chautems & M. Perret 99-046 (G)	Cultivated MP-52 (Brazil)	JX195779	JX195862	*KT958471*	*KT958358*	*KT958300*	*KT958411*	1	0	0
Nematanthus pycnophyllus Chautems, T. Lopes & M. Peixoto	A. Chautems & M. Perret 02-010 (G)	Cultivated CJBG AC-2702 (Brazil)	JX195780	JX195863	*KT958472*	*KT958359*	*KT958301*	*KT958412*	1	1	1
Nematanthus sericeus (Hanst.) Chautems	A. Chautems & M. Perret 99-018 (G)	Cultivated CJBG AC-1171 (Brazil)	JX195781	JX195864	*KT958473*	*KT958360*	*KT958302*	*KT958413*	1	1	0
Nematanthus serpens (Vell.) Chautems	A. Chautems & M. Perret 11-001 (G)	Cultivated CJBG AC-3509 (Brazil)	JX195782	JX195865	*KT958474*	*KT958361*	*KT958303*	*KT958414*	1	1	0
Nematanthus strigillosus (Mart.) H.E.Moore	A. Chautems & M. Perret 11-101 (G)	Cultivated CJBG AC-3511 (Brazil)	JX195783	JX195866	*KT958475*	*KT958362*	*KT958304*	*KT958415*	1	1	0
Nematanthus teixeiranus (Handro) Chautems	A. Chautems & M. Perret 07-407 (G)	Cultivated CJBG AC-1127 (Brazil)	JX195785	JX195868	*KT958476*	*KT958363*	*KT958305*	*KT958416*	1	1	0
Nematanthus tessmannii(Hoehne) Chautems	A. Chautems & M. Perret 07-408 (G)	Cultivated CJBG AC-1131 (Brazil)	JX195786	JX195869	*KT958477*	*KT958364*	*KT958306*	*KT958417*	1	1	1
Nematanthus villosus (Hanst.) Wiehler	A. Chautems & M. Perret 99-041 (G)	Cultivated CJBG AC-1108 (Brazil)	AJ439825	GQ383608	*KT958478*	*KT958365*	*KT958307*	*KT958418*	1	1	1
Nematanthus wettsteinii (Fritsch) H.E.Moore	A. Chautems & M. Perret 10-102 (G)	Cultivated CJBG AC-1321 (Brazil)	JX195787	JX195870	*KT958479*	*KT958366*	*KT958308*	*KT958419*	1	1	0
Nematanthus wiehleri Chautems & M. Peixoto	A. Chautems & M. Perret 12-101 (G)	Cultivated CJBG AC-1482 (Brazil)	JX195788	JX195871	*KT958480*	*KT958367*	*KT958309*	*KT958420*	1	0	0
Neomortonia nummularia (Hanst.) Wiehler	A. Chautems & M. Perret 09-101 (G)	CultivatedCJBG s.n. (Central America & Andes)	JX195791	JX195874	*KT958482*	*KT958369*	*KT958311*	*KT958422*	NA	NA	NA
Sinningia schiffneri Fritsch	A. Chautems & M. Perret 97-010 (G)	Cultivated CJBG AC-1340 (Brazil)	AJ439745	GQ383621	*KT958483*	*KT958370*	*KT958312*	*KT958423*	NA	NA	NA

Table S2. Pollinator, locality and source of information for *Nematanthus* species

Species	Locality	*Nematanthus*-lineage	Visitors	Reference
N. brasiliensis	Mangaratiba, Reserva Ecológica Rio das Pedras, RJ (30m)	*Nematanthus-A*	[a]*Phaetornis rubber*[*]<br>*Ramphodon naevius*[*]	(SanMartin-Gajardo and Vianna, 2010)
N. fissus	Caraguatatuba, SP (50-100m)	*Nematanthus-A*	*Phaetornis rubber*[*]<br>*Thalurania glaucopis*[+]	(Buzato et al., 2000)
N. fluminensis	Caraguatatuba SP (50-100m)	*Nematanthus-A*	*Ramphodon naevius*[*]<br>[a]*Phaetornis rubber*[*]	(Buzato et al., 2000)
N. fornix	Campos do Jordão, SP (1400-1600m)	*Nematanthus-B*	*Phaetornis eurynome*[*]<br>*Leucochloris albicollis*[+]<br>*Stephanoxis lalandi*[+]	(Buzato et al., 2000)
N. fornix	Itatiaia National Park (900-1200m)	*Nematanthus-B*	*Phaethornis eurynome*[*]<br>*Phaethornis squalidus*[*]<br>*Thalurania glaucopis*[+]	Wolowski et al. 2013
N. fritschii	Estação Ecológica Estadual Juréia-Itatins, SP (800-900m)	*Nematanthus-A*	*Ramphodon naevius*[*]	(Franco and Buzato, 1992)
N. gregarius	Boracéia forest reserve, SP (800-900m)	*Nematanthus-B*	*Leucochloris albicollis*[+]<br>*Clytolaema rubricauda*[+]<br>*Thalurania glaucopis*[+]	Snow and Snow 1986
N. sericeus	Cunha, SP (1000-1100m)	*Nematanthus-B*	*Leucochloris albicollis*[+]	(Buzato et al., 2000)
N. strigilosus	Serra da Piedade, MG (1400-1740m)	*Nematanthus-B*	*Colibri serriostris*[+]<br>*Chlorostilbon aureoventris*[+]	Vasconcelos & Lombardi 2001
N. strigilosus	Serra do Caraça, MG (1200-2020m)	*Nematanthus-B*	*Thalurania glaucopis*[+]<br>*Amazilia lactea*[+]	Vasconcelos & Lombardi 2001

N. lanceolatus	Itatiaia National Park (900-1200m)	*Nematanthus-A*	*Phaethornis eurynome*[*]<br>*Phaethornis squalidus* [*]	Wolowski et al. 2013
N. crassifolius	Itatiaia National Park (900-1200m)	*Nematanthus-A*	*Phaethornis eurynome*[*]	Wolowski et al. 2013

[a] nectar robber

[*] *Phaethornithinae*

[+] *Trochilinae*

Table S3. Mean values for floral traits used for the principal component analyses. Abbreviations in figure S1.

Species	obs	DHL	DOH	DVL	DOV	DTH	DTV	LTU	DRH	DRV	DNH	DNV	LAN	LST
Codonanthe carnosa	8.0	16.9	7.9	15.4	6.7	5.6	5.3	16.7	2.9	2.9	2.9	3.3	11.8	8.8
Codonanthe cordifolia	3.0	9.7	6.3	8.0	5.4	4.9	4.8	11.1	3.1	3.1	3.1	3.1	8.2	8.0
Codonanthe corniculata	5.0	10.7	5.9	10.4	4.7	6.2	5.4	22.2	2.9	2.4	2.9	3.0	17.0	10.4
Codonanthe devosiana	11.0	14.2	6.6	14.0	5.3	4.7	3.9	10.4	2.6	2.3	2.7	2.7	8.9	6.8
Codonanthe elegans	6.0	17.2	9.1	17.9	8.5	7.9	7.5	33.6	3.1	3.2	3.6	4.5	17.6	21.0
Codonanthe gracilis	9.0	16.4	6.8	16.8	5.9	6.9	7.4	13.8	3.4	3.5	3.8	4.6	10.5	10.6
Codonanthe macradenia	5.0	13.6	8.8	9.9	6.7	9.0	8.1	30.5	3.7	3.0	3.0	3.9	17.1	23.4
Codonanthe serrulata	5.0	11.9	6.1	11.2	6.1	6.4	6.9	15.9	2.8	2.8	3.0	3.5	15.7	11.6
Codonanthe uleana	4.0	14.2	7.1	16.0	7.2	6.6	5.4	25.8	2.8	2.6	2.6	4.4	18.3	20.0
Codonanthe venosa	7.0	12.7	6.5	12.7	5.8	5.1	4.7	16.4	2.6	2.4	2.7	3.5	14.5	11.2
Nematanthus albus	4.0	33.3	15.2	33.0	17.3	18.0	18.0	48.8	4.7	6.1	5.7	8.5	31.1	30.0
Nematanthus australis	11.0	5.6	3.5	5.2	3.4	6.6	8.5	17.3	2.6	4.0	3.2	4.0	14.0	12.1
Nematanthus bradei	1.0	4.4	3.8	4.4	3.5	9.1	11.3	16.6	2.6	2.1	3.0	3.3	17.8	12.4
Nematanthus brasiliensis	2.0	23.3	11.2	34.4	19.7	8.8	18.1	46.5	5.0	6.1	7.7	11.6	58.2	58.7
Nematanthus corticola	3.0	17.1	6.7	11.6	14.0	5.9	15.5	36.8	3.1	4.0	3.4	5.6	35.8	38.7
Nematanthus crassifolius	12.0	15.0	9.9	26.2	21.3	9.2	20.3	53.3	3.3	4.7	4.7	7.8	49.3	59.3
Nematanthus fissus	2.0	5.2	3.4	5.4	4.0	6.6	8.3	28.1	2.3	3.5	3.5	4.5	27.2	23.8
Nematanthus fluminensis	16.0	12.7	8.2	20.7	15.7	7.6	15.2	52.4	3.8	5.0	4.9	9.3	47.0	59.8
Nematanthus fornix	4.0	5.9	4.8	5.1	3.9	11.0	12.4	25.0	2.9	3.8	3.4	4.3	19.0	19.8
Nematanthus fritschii	9.0	9.0	6.1	11.7	9.4	10.5	14.0	43.2	3.4	4.4	4.2	7.6	40.3	45.8
Nematanthus gregarius	10.0	4.8	3.6	4.3	3.5	9.0	11.2	22.5	2.9	3.5	3.5	3.8	19.8	14.9
Nematanthus hirtellus	5.0	9.3	5.5	9.1	6.5	7.3	8.3	25.4	4.0	5.5	4.8	7.5	18.3	24.5
Nematanthus jolyanus	2.0	6.8	4.5	6.9	5.3	7.3	11.8	32.2	3.2	4.1	4.0	5.8	26.0	26.9
Nematanthus maculatus	7.0	6.3	4.8	6.8	5.4	7.4	10.8	30.3	3.1	3.8	3.8	7.0	25.4	25.3
Nematanthus monanthos	2.0	5.8	4.7	6.0	5.4	8.1	12.1	35.8	2.6	4.0	3.7	6.2	28.2	25.9
Nematanthus punctatus	1.0	41.9	21.2	36.3	15.5	19.8	17.9	49.6	4.6	6.8	5.9	6.7	37.9	32.7
Nematanthus pycnophyllus	4.0	4.7	3.5	5.7	4.6	5.1	8.0	31.4	2.2	2.6	3.2	3.8	27.9	28.0
Nematanthus sericeus	3.0	7.0	4.8	6.5	4.2	11.9	13.9	26.9	3.1	3.3	3.3	4.0	27.7	21.6
Nematanthus serpens	5.0	5.0	3.6	4.8	3.3	7.9	10.7	19.8	2.3	2.8	2.8	3.5	19.1	17.0
Nematanthus strigillosus	7.0	6.4	4.0	6.1	4.1	9.0	11.5	25.1	3.2	3.7	4.1	4.7	25.5	19.4
Nematanthus teixeiranus	3.0	5.1	3.8	4.3	3.5	8.1	11.1	23.2	2.4	3.1	3.1	3.9	19.0	19.7
Nematanthus tessmannii	12.0	5.8	4.4	5.4	4.1	8.9	10.5	25.3	3.4	3.5	4.0	6.1	22.6	20.4
Nematanthus villosus	9.0	5.5	4.5	5.2	4.4	7.1	8.1	22.4	2.1	3.2	2.9	5.6	19.9	19.6
Nematanthus wettsteinii	2.0	3.9	3.6	3.0	2.8	6.0	9.4	21.5	2.6	3.6	3.3	3.7	13.6	12.3
Nematanthus wiehleri	2.0	30.1	14.9	30.0	14.8	20.5	18.7	48.9	5.6	7.4	7.2	9.3	37.3	29.7
Codonanthe mattos-silvae	3.0	16.2	8.3	15.8	6.9	8.2	6.4	23.7	4.1	4.6	3.9	4.6	16.1	19.0
Codonanthe caribaea	3.0	21.9	9.6	22.0	8.1	8.2	6.4	23.5	2.8	3.0	2.3	3.3	14.5	15.4
Codonanthopsis ulei	2.0	12.9	7.9	15.0	8.9	7.5	8.5	20.7	2.5	2.5	2.0	8.3	14.7	16.5

Table S4. List of the herbaria where specimens have been examined for occurence data. Acronyms follow Index Herbariorum (http://sweetgum.nybg.org/ih/).

Abbreviation	Herbarium name
B	Botanischer Garten und Botanisches Museum Berlin-Dahlem, Zentraleinrichtung der Freien Universität Berlin
BHCB	Universidade Federal de Minas Gerais
BM	British Museum of Natural History
BOTU	Herbario Irina Delanova Gemtchajnicov
BR	National Botanic Garden of Belgium
CEPEC	Herbario do Centro de Pesquisas do Cacau
CESJ	Herbario Leopoldo Krieger
E	Royal Botanic Garden, Edinburgh
ESA	Herbario Da Escola Superior de Agricultura Luiz de Queiroz
F	Herbarium Botany Department, Field Museum of Natural History
G	Conservatoire et Jardin botaniques de la Ville de Genève
GFJP	Herbário Guido F. J. Pabst, Faculdade Redentor
GH	The Gray Herbarium
GUA	Herbário Alberto Castellanos, FEEMA, Serviço de Ecologia Aplicada, DIVEA, DEP, FEEMA
HAS	Herbário Alarich Rudolf Holger Schultz, Fundação Zoobotânica do Rio Grande do Sul
HBR	Herbário Barbosa Rodrigues, Universidade Federal de Santa Catarina
HPL	Herbário, Instituto Plantarum de Estudos da Flora Ltda
HRCB	Herbário Rioclarense, Instituto de Biociências, Universidade Estadual Paulista
HUEFS	Herbário, Departamento de Ciências Biológicas, Universidade Estadual de Feira de Santana
IAC	Herbário, Centro de Recursos Genéticos Vegetais e Jardim Botánico, Instituto Agronômico de Campinas
IAN	Herbário, Laboratório de Botânica, Embrapa Amazônia Oriental
IAS	Indiana Academic of Science
ICN	Herbário ICN, Departamento de Botânica
INPA	Herbário, Coordenação de Pesquisas em Botânica, Instituto Nacional de Pesquisas da Amazônia
JPB	Herbário Lauro Pires Xavier
K	Herbarium, Royal Botanic Gardens
M	Herbarium, Botanische Staatssammlung München
MBM	Herbário, Museu Botânico Municipal
MBML	Herbário, Museu de Biologia Mello Leitão
MG	Herbário, Museu Paraense Emílio Goeldi
MO	Herbarium, Missouri Botanical Garden
NY	New York Botanical Garden
P	Muséum National d'Histoire Naturelle
PACA	Herbário Anchieta, Instituto Anchietano de Pesquisas/UNISINOS
PEL	Herbário,Universidade Federal de Pelotas
R	Herbário, Departamento de Botânica, Museu Nacional
RB	Herbário, Instituto de Pesquisas, Jardim Botânico do Rio de Janeiro
RU	Herbarium, Agricultural Botany Department, University of Reading
S	Herbarium, Swedish Museum of Natural History
SEL	Herbarium, Marie Selby Botanical Gardens
SP	Herbário,Instituto de Botânica
SPSF	Herbário Dom Bento José Pickel, Divisão de Dasonomia, Seção de Madeiras e Produtos Florestais
UB	Herbário, Universidade de Brasília
UEC	Herbário, Instituto de Biologia,IB, Universidade Estadual de Campinas
UFP	Herbário, Universidade Federal de Pernambuco
UPCB	Herbário, Universidade Federal do Paraná
US	United States National Herbarium, Smithsonian Institution
VIC	Herbário, Universidade Federal de Viçosa
VIES	Central Herbarium UFES, Federal University of Espírito Santo
W	Herbarium, Naturhistorisches Museum Wien
WAG	Nationaal Herbarium Nederland, Wageningen University branch, Wageningen University
WU	Herbarium, Faculty Center Botany, Department of Plant Systematics and Evolution, Universität Wien
Z	Joint Herbarium of the University of Zurich and the ETH Zurich

Table S5. PC loadings for morphological and climatic preferences. Description of floral morphology traits in legend of table S2.

Floral morphology			Climatic variables		
Trait	PC1	PC2	Trait	PC1	PC2
DHL	-0.245	-0.4611	Mean annual Temperature	0.8634	-0.0221
DOH	-0.261	-0.3972	Mean diurnal range	-0.1525	0.3717
DVL	-0.2837	-0.2485	Isothermality	0.8417	0.2029
DOV	-0.3023	0.0141	Temperature Seasonality	-0.8939	-0.205
DTH	-0.2518	-0.2276	Max Temperature warmest month	0.7341	-0.0214
DTV	-0.2834	0.2393	Min Temperature coldest month	0.8974	-0.0448
LTU	-0.2964	0.2212	Annual Range Temperature	-0.8103	0.0541
DRH	-0.2803	-0.2169	Mean Temperature wettest	0.6648	-0.1729
DRV	-0.2895	-0.0512	Mean Temperature driest	0.9166	0.0331
DNH	-0.2981	0.0468	Mean Temperature warmest	0.7347	-0.1172
DNV	-0.2792	0.2209	Mean Temperature coldest	0.9094	0.0338
LAN	-0.2719	0.377	Annual precipitation	0.6345	0.0668
LST	-0.2555	0.4085	Precipitation wettest month	0.5838	0.31
			Precipitation driest month	0.2853	-0.3951
			Precipitation seasonality	-0.0432	0.6337
			Precipitation wettest month	0.5802	0.2775
			Precipitation driest month	0.3482	-0.3728
			Precipitation warmest month	-0.1408	-0.0159
			Precipitation coldest month	0.6944	0.0165
			Altitude	-0.473	0.3528

Table S6. AICc values for the complete set of OU models. Regimes in pollinator type and geography are explained in the main text. Model parameters: α = selection strength, σ^2 = rate of evolution, and θ = state means. OUM assumes different θ and a single α and σ^2 acting on the selective regimes. OUMV with different θ and σ^2, OUMA with multiple α, and OUMVA with multiple α and σ^2 per selective regime. The preferred OU model for floral size was OUMV and floral shape the OUMVA, with regimes according to pollination syndromes. For mean and seasonality in temperature the model OUM and precipitation seasonality model OUM, with regimes determined by geography, were selected.

**Models**	**Pollination syndrome**				**Geography**			
	OUM AICc	OUMV AICc	OUMA AICc	OUMVA AICc	OUM AICc	OUMV AICc	OUMA AICc	OUMVA AICc
Morphology PC1	189.2001	172.9199	189.5848	173.1399	195.8302	187.4603	192.4959	139.0627
Morphology PC2	88.0996	90.609	83.7823	79.5401	108.8082	103.3185	109.6878	106.0105
Climate PC1	134.387	136.9541	136.9557	142.1494	124.4231	126.9841	125.4141	126.2922
Climate PC2	102.8038	105.3339	105.2334	107.1134	100.5768	100.6149	101.8	102.9673

Table S7. Model fit comparison correcting for measurement error (ME). Bold indicate the selected model. Asterisk indicates that the error was not estimated for this type of model, but fixed with the error estimated under a single BM model.

Model	AICc	AICc incorporate error	Estimated ME
MORPHOLOGY PC1			
BM	190.9718	190.4220	1.4296
OU single	187.7321	190.2145	0.3425
EB (ac)	193.3461	192.9283	0.8944
EB (dc)	187.7321	190.2145	0.3429
OUWie_OUM	189.2001	191.7122	0.5557
OUWie_OUMV	172.9199	175.1343	0.3681
OUWie_OUMA	189.5848	188.0949	2.2075
OUWie_OUMVA	173.1399	172.0341	0.4584
Multiple rates	**152.2585**	**160.0260**	*
MORPHOLOGY PC2			
Model	AICc	AICc incorporate error	Estimated ME
BM	104.3669	106.7198	0.0000
OU single	106.6641	109.1489	0.0000
EB (ac)	106.7412	109.2261	0.0000
EB (dc)	106.6641	109.1489	0.0000
OUWie_OUM	88.0996	91.8741	0.4759
OUWie_OUMV	90.6090	94.6933	0.4646
OUWie_OUMA	83.7823	91.1330	0.3894
OUWie_OUMVA	**79.5401**	**83.6943**	0.1677
Multiple rates	102.3854	102.3854	*
CLIMATIC PREFERENCES PC1			
Model	AICc	AICc incorporate error	Estimated ME
BM	134.5776	134.3708	0.5960
OU single	135.6864	136.8080	0.5960
EB (ac)	136.9015	139.3226	0.0000
EB (dc)	136.5797	136.8080	0.5960
OUWie_OUM	**124.4231**	**125.0787**	0.8193
OUWie_OUMV	126.9841	127.7904	0.8193
OUWie_OUMA	125.4141	127.2266	0.8086
OUWie_OUMVA	126.2922	130.0933	0.8086
Multiple rates	130.7428	131.8210	*
CLIMATIC PREFERENCES PC2			
Model	AICc	AICc incorporate error	Estimated ME
BM	115.0848	100.7321	0.6284
OU single	100.5829	102.5151	0.5514
EB (ac)	117.4086	103.1693	0.6284
EB (dc)	100.5829	102.5151	0.5514
OUWie_OUM	100.5768	102.8283	0.2988
OUWie_OUMV	100.6149	106.6520	0.4666
OUWie_OUMA	101.8000	106.6175	0.4673
OUWie_OUMVA	102.9673	105.5555	0.0855
Multiple rates	**95.1331**	**95.0651**	*

Figure S1. Floral measurements used for the principal component analysis. A-C: *Nemantanthus fritschii,* D: *Codonanthe devosiana*. Photographs used with permission from Manuel Faustino. Abbreviations as follows: DHL: corolla horizontal diameter; DVL: corolla vertical diameter; DOH: horizontal diameter of the corolla opening; DOV: corolla vertical diameter at the corolla opening; DTH: Maximum horizontal tube diameter; DTV: Maximum vertical tube diameter; LTU: Dorsal tube length; DRH: Horizontal diameter of the tube constriction anterior to the nectar chamber; DRV: Vertical diameter of the tube constriction anterior to the nectar chamber; DNH: Horizontal diameter of the nectar chamber; DNV: Vertical diameter of the nectar chamber; LAN: Length from the anther base to the ovary; LST: Length from the stigma base to the ovary.

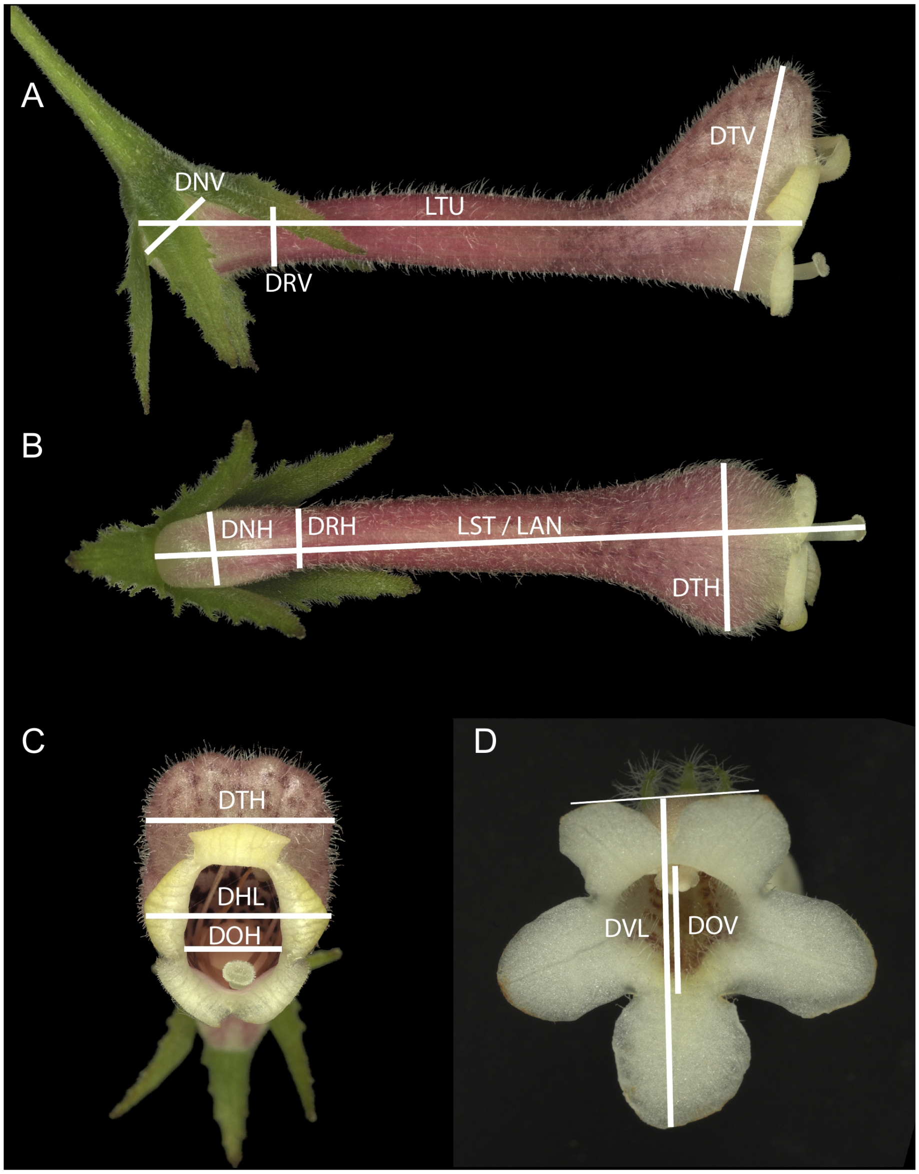

Figure S2. Phylogenetic reconstruction with outgroup species. Blue bars correspond to the 95% highest posterior density. Values above branches are Bayesian posterior probabilities. Names on bars denote tribes of the *Gesnerioideae* subfamily.

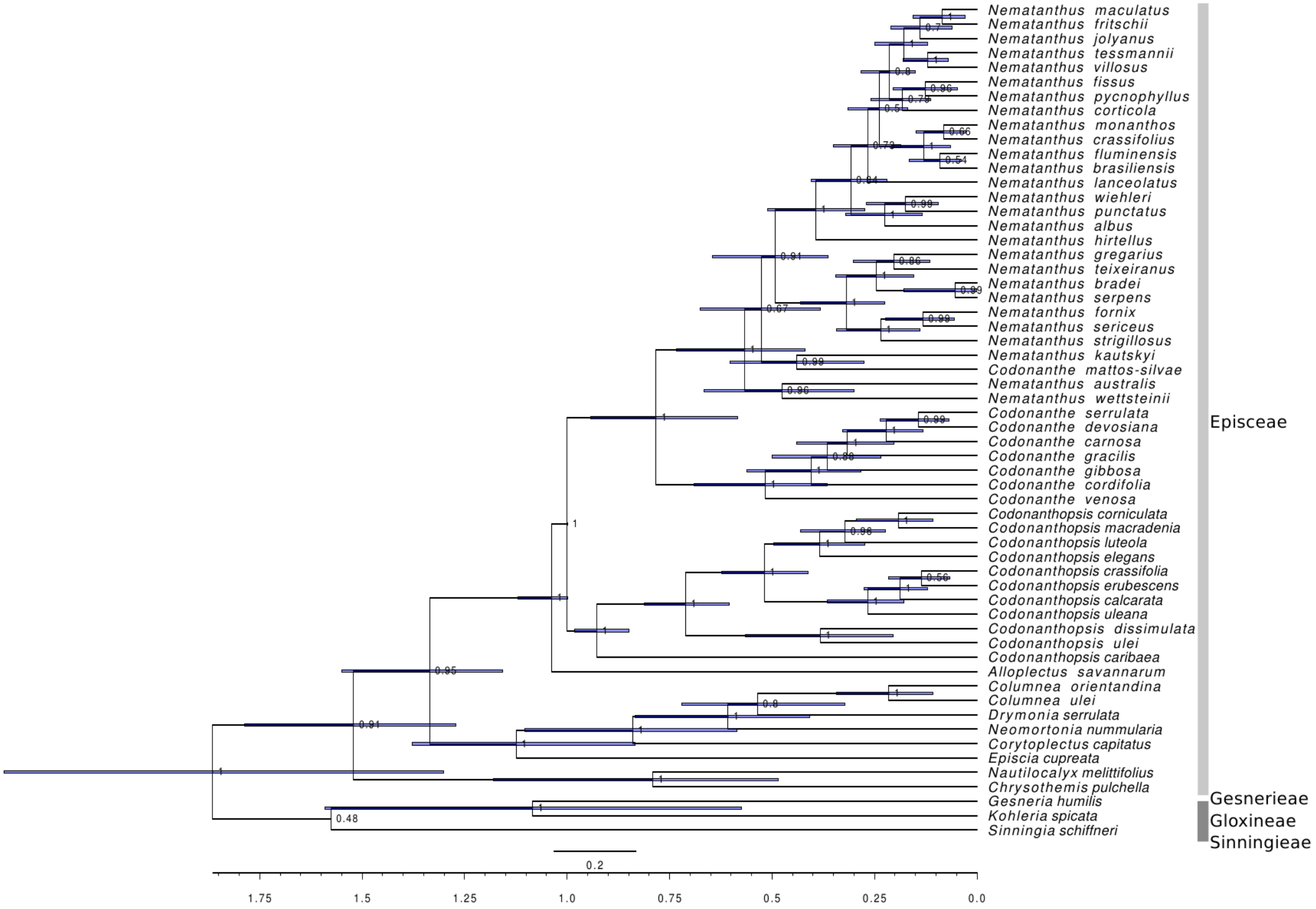

Figure S3. Ancestral state reconstructions. Maximum likelihood ancestral state reconstruction (corHMM R package), details of states in text. ER and ARD, equal rates and all rates different models, respectively.

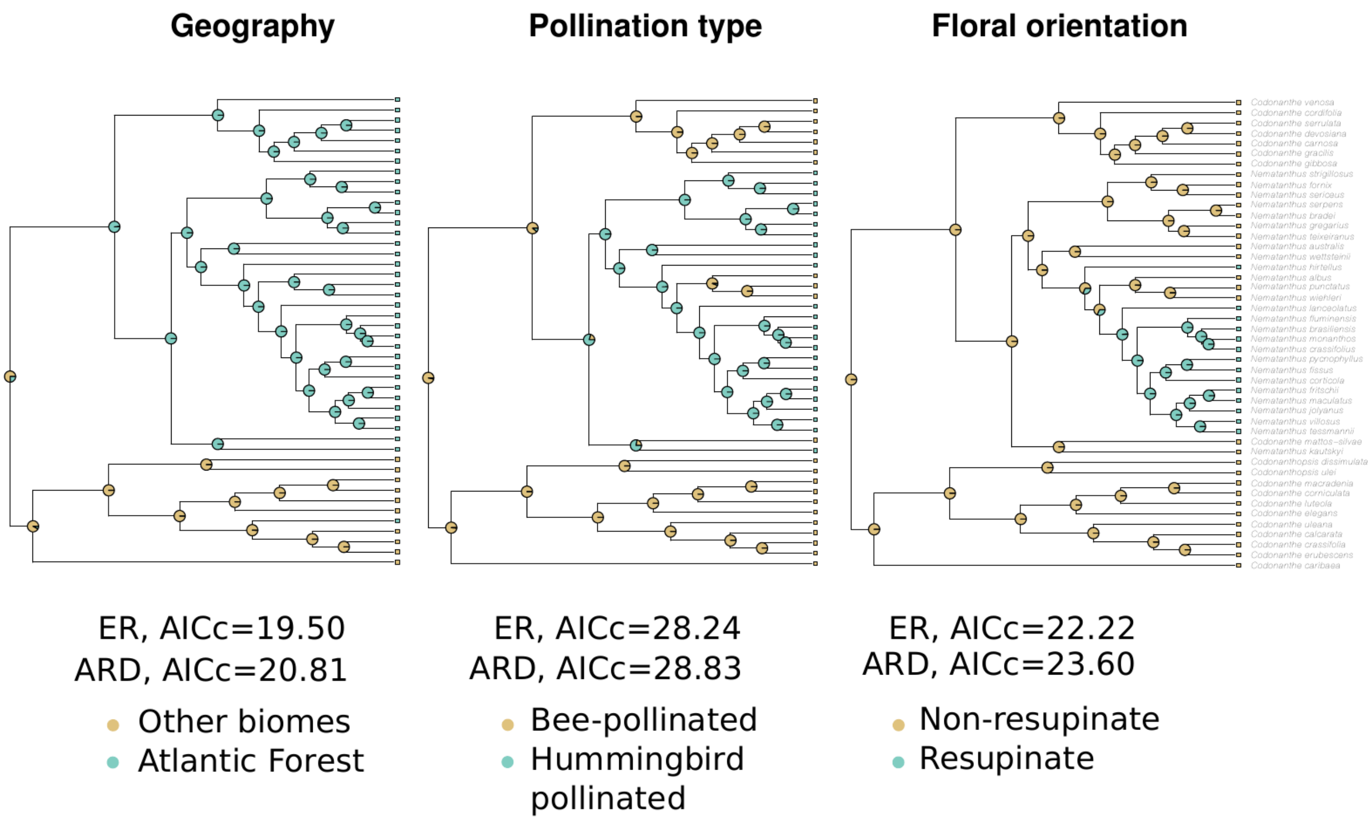

Figure S4. Panel a) Principal component analysis of 38 CCN species based on 13 floral traits. Colors correspond to the clades according to the legend. Species names: 1, *N. punctatus;* 2, *N. Albus*; 3, *N. wiehleri*; 4, *N. fluminensis*; 5, *N. crassifolius*; 6, *N. brasiliensis*; 7, *N. corticola*; 8, *N. fritschii*; 9, *N. tessmannii;* 10, *N. villosus*; 11, *N. maculatus;* 12, *N. fissus*; 13, *N. pycnophyllus*; 14 *N. monanthos*; 15, *N. jolyanus*; 16, *N. hirtellus*; 17, *N. gregarius*; 18, *N. bradei*; 19, *N. sericeus*; 20, *N. teixeiranus;* 21, *N. strigillosus*; 22, *N. serpens*; 23, *N. wettsenii;* 24, *N. australis*; 25, *C. venosa;* 26, *C. cordifolia;* 27, *C. devosiana*; 28, *C. serrulata*; 29*, C. carnosa*; 30, *C. gracilis;* 31, *C. uleana;* 32, *C. caribaea;* 33, *C. macradenia;* 34, *C. elegans*; 35, *Codonanthopsis ulei*; 36, *N. fornix;* 37, *C. mattos-silvae*; 38, *C. corniculata*. Panel b) Principal component analysis of climatic preferences in 43 CCN species, and geographical distribution by subclade. The distribution map was produced in this study. Species names: 1 *Codonanthe calcarata*, 2 *Codonanthe caribaea,* 3 *Codonanthe carnosa*, 4 *Codonanthe cordifolia,* 5 *Codonanthe crassifolia,* 6 *Codonanthe devosiana,* 7 *Codonanthe erubescens*, 8 *Codonanthe gibbosa,* 9 *Codonanthe gracilis,* 10 *Codonanthe luteola,* 11 *Codonanthe macradenia,* 12 *Codonanthe mattos.silvae,* 13 *Codonanthe serrulata,* 14 *Codonanthe uleana*, 15 C*odonanthe venosa,* 16 *Codonanthopsis dissimulat*a, 1*7 Codonanthopsis ulei*, 18 *Nematanthus albus*, 19 *Nematanthus australis,* 20 *Nematanthus bradei,* 21 *Nematanthus brasiliensis,* 22 *Nematanthus corticola*, 23 *Nematanthus crassifolius,* 24 *Nematanthus fissus,* 25 *Nematanthus fluminensis,* 26 *Nematanthus fornix,* 27 *Nematanthus fristchi,* 28 *Nematanthus gregarius,* 29 *Nematanthus hirtellus*, 30 *Nematanthus jolyanus*, 31 *Nematanthus kautskyi,* 32 *Nematanthus lanceolatus*, 33 *Nematanthus maculatus,* 34 *Nematanthus monanthos,* 35 *Nematanthus punctatus,* 36 *Nematanthus pycnophyllus* , 37 *Nematanthus sericeus,* 38 *Nematanthus strigillosus,* 39 *Nematanthus teixeiranus,* 40 *Nematanthus tessmannii,* 41 *Nematanthus villosus,* 42 *Nematanthus wettsteinii,* 43 *Nematanthus wiehleri.* Photographs 3,6,17-20,23,26,27,30,32 and 34 by Mauro Peixoto, photographs 2,4,5,7,9,10-16,22,29,31,33,35 by John Clark, photographs 1,8, 28 by Alain Chautems, and photograph 25 by Bernard Renaud. All photographs were used under permission requests from the owners.

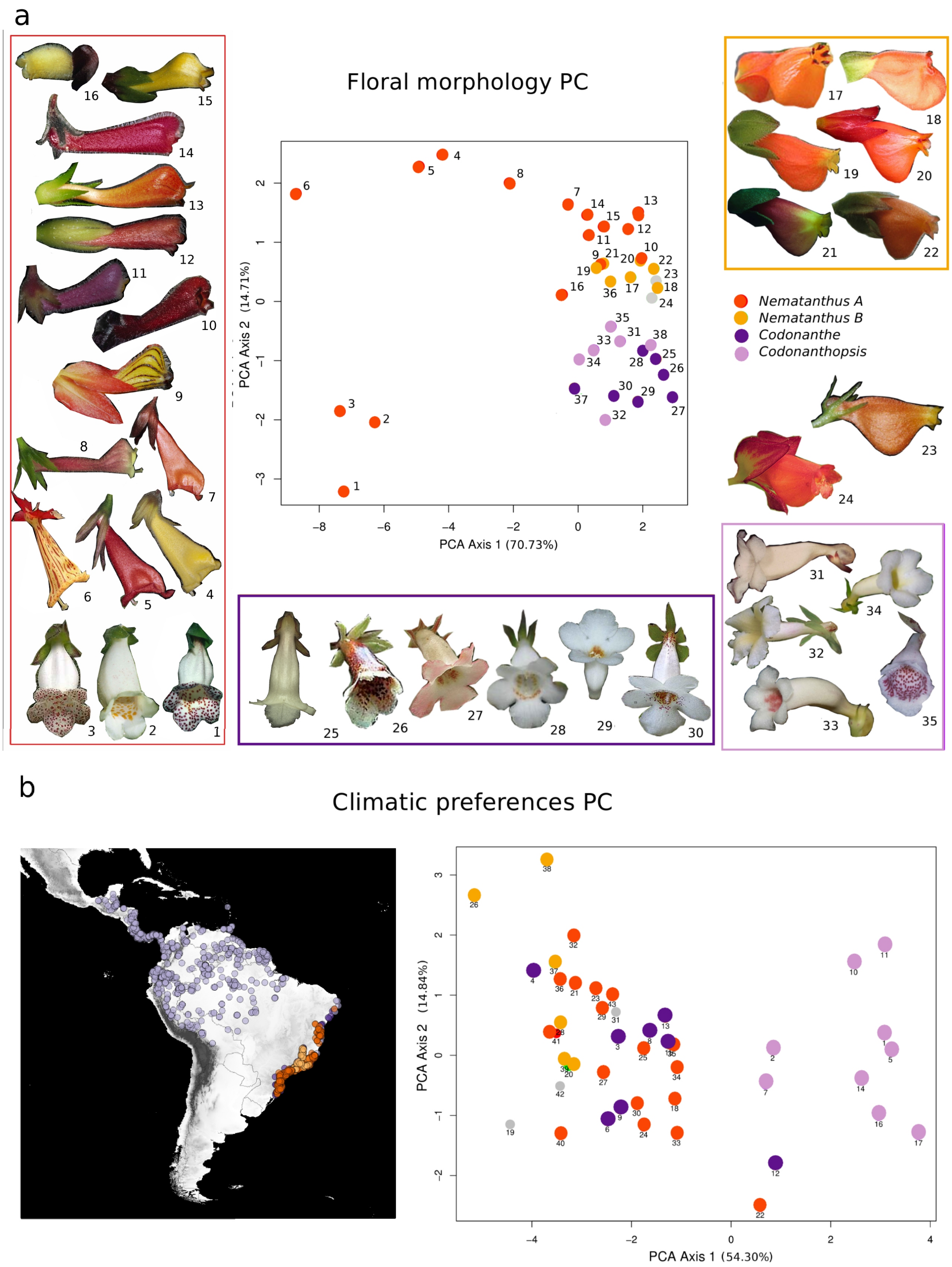
a
Floral morphology PC
PCA Axis 1 (70.73%)
PCA Axis 2 (14.71%)
Nematanthus A
Nematanthus B
Codonanthe
Codonanthopsis
b
Climatic preferences PC
PCA Axis 1 (54.30%)
PCA Axis 2 (14.84%)

Figure S5. Comparison of phenotypic space and multi-rates BM model from raw (upper panel) and log-transformed (lower panel) morphological measurements.

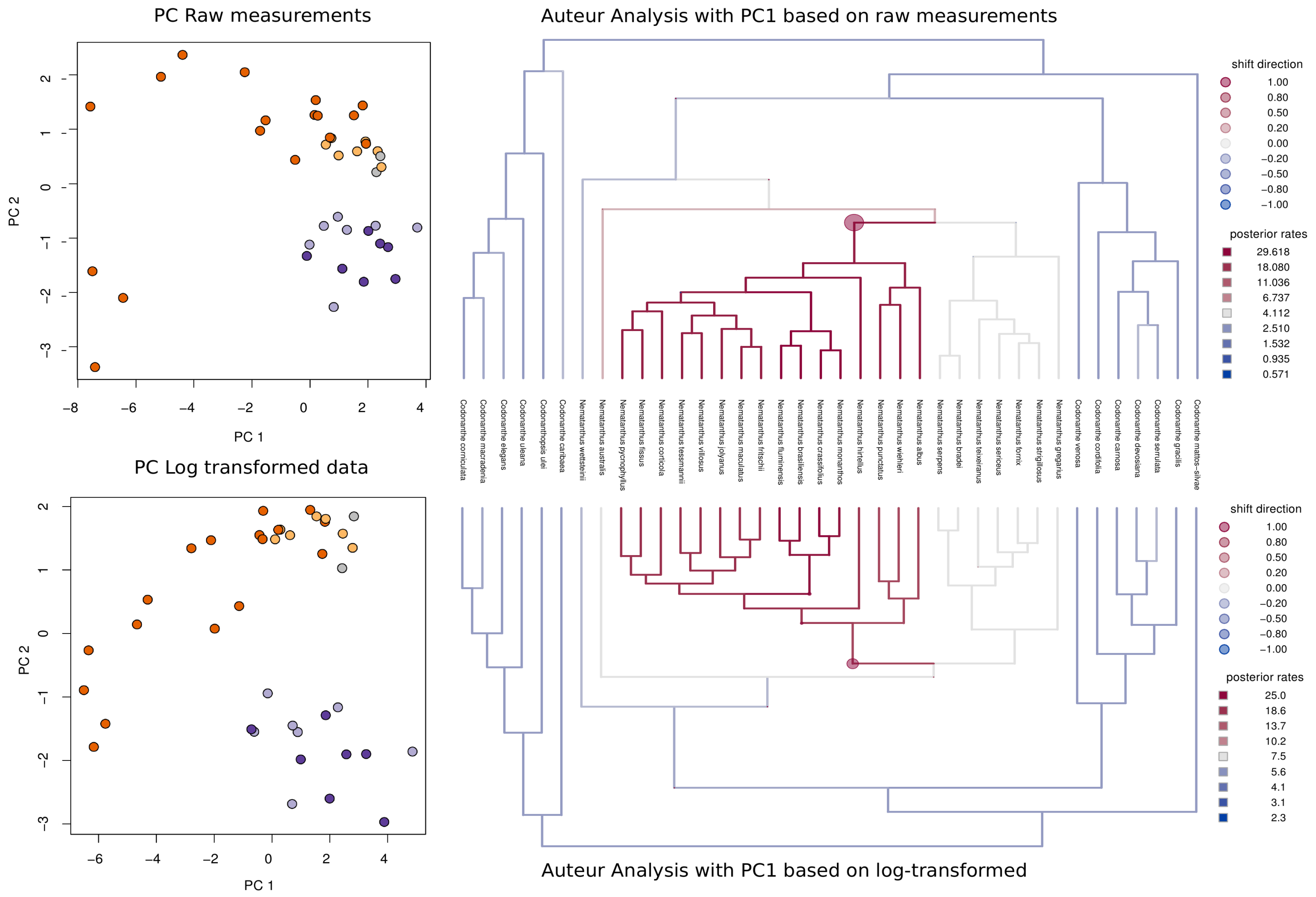

Figure S6. Credibility tree from MrBayes with outgroup sequences included. Numbers on nodes are posterior probabilities for each clade.

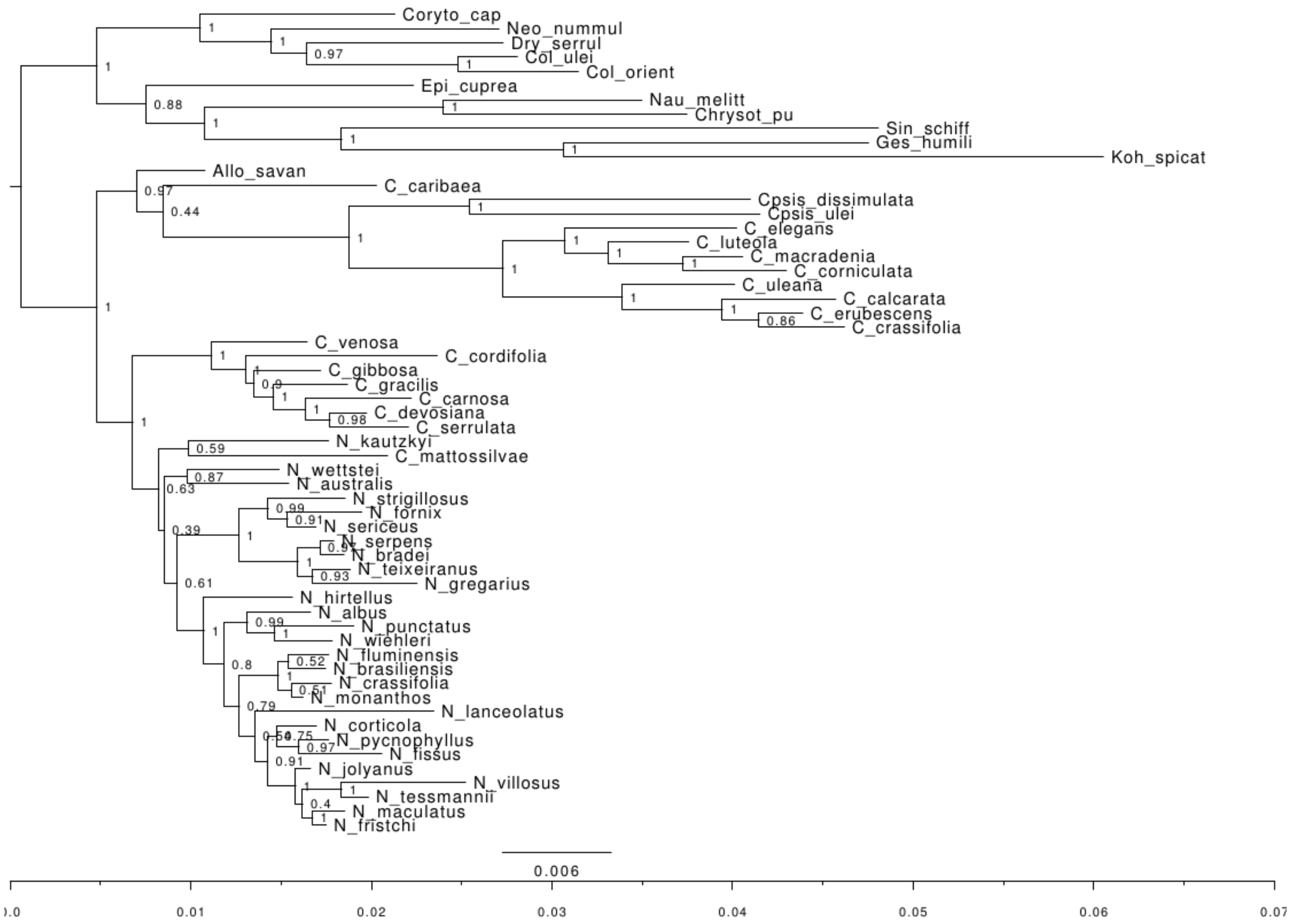

References

Beaulieu, J. M., B. C. O'Meara, and M. J. Donoghue. 2013. Identifying hidden rate changes in the evolution of a binary morphological character: the evolution of plant habit in campanulid angiosperms. Systematic Biology. 62:725–37.

Buzato, S., M. Sazima, and I. Sazima. 2000. Hummingbird-pollinated floras at three Atlantic forest sites. Biotropica 32:824– 841.

Chautems, A. 1988. Révision taxonomique et possibilités d'hybridations de *Nematanthus* Schrader (Gesneriaceae), genre endémique de la forêt côtière brésilienne. Dissertationes Botanicae. 112:1–226.

Chautems, A. 2009. Gesneriaceae. Pp. 285–288 *in* J. Stehmann, R. Forzza, A. Salino, M. Sobral, D. Costa, and L. Kamino, eds. Plantas da floresta Atlântica. Rio de Janeiro.

Chautems, A., and M. Perret. 2013. Redefinition of the neotropical genera *Codonanthe* (Mart.) Hanst, and *Codonanthopsis* Mansf. (Gesneriaceae). Selbyana 31:143–157.

Clark, J. L., M. M. Funke, A. M. Duffy, and J. F. Smith. 2012. Phylogeny of a Neotropical clade in the Gesneriaceae: more tales of convergent evolution. International Journal of Plant Sciences. 173:894–916.

Drummond, A. J., M. A. Suchard, D. Xie, and A. Rambaut. 2012. Bayesian Phylogenetics with BEAUti and the BEAST 1.7. Molecular Biology and Evolution. 29:1969–1973.

Franco, M. A., and S. Buzato. 1992. Biologia floral de *Nematanthus fritschii* (Gesneriaceae). Revista Brasileira de Biologia. 52:661–666.

Katoh, M., and M. Kuma. 2002. MAFFT: a novel method for rapid multiple sequence alignment based on fast Fourier transform. Nucleic Acids Research. 30:3059–3066.

Paradis, E., J. Claude, and K. Strimmer. 2004. APE: Analyses of Phylogenetics and Evolution in R language. Bioinformatics 20:289–290.

Penn, O., E. Privman, H. Ashkenazy, G. Landan, D. Graur, and T. Pupko. 2010. GUIDANCE: a web server for assessing alignment confidence scores. Nucleic Acids Research. 38:23–28.

Perret, M., A. Chautems, A. O. De Araujo, and N. Salamin. 2013. Temporal and spatial origin of Gesneriaceae in the New World inferred from plastid DNA sequences. Botanical Journal of the Linnean Society. 171:61–79.

Rambaut, A., and A. J. Drummond. 2007. Tracer v1.4.

Revell, L. J. 2012. phytools: an R package for phylogenetic comparative biology (and other things). Methods Ecoloy and Evolution. 3:217–223.

Ronquist, F., M. Teslenko, P. van der Mark, D. Ayres, A. Darling, S. Hohna, B. Larget, L. Liu, M. A. Suchard, and J. Huelsenbeck. 2012. MrBayes 3.2: Efficient bayesian phylogenetic inference and model choice across a large model space. Syst. Biol. 61:539–542.

Rosinni, J. and Chautems, A. 2007. Codonanthe gibossa Rossini & Chautems (Gesneriaceae), a new species from the State of Espírito Santo, Brazil. Candollea. 62: 215-220.

Sanmartin-Gajardo, I., and J. R. . Viana. 2010. Pollination of *Nematanthus brasiliensis*: An epiphytic Gesneriaceae endemic to the Southeastern Atlantic Forest of Brazil. Selbyana 30:216–220.

Sauquet, H., S. Y. W. Ho, M. a Gandolfo, G. J. Jordan, P. Wilf, D. J. Cantrill, M. J. Bayly, L. Bromham, G. K. Brown, R. J. Carpenter, D. M. Lee, D. J. Murphy, J. M. K. Sniderman, and F. Udovicic. 2012. Testing the impact of calibration on molecular divergence times using a fossil-rich group: the case of Nothofagus (Fagales). Systematic Biology. 61:289–313.

Snow, D. W., and B. K. Snow. 1986. Feeding ecology of hummingbirds in the Serra do Mar , southeastern Brazil. Rev. Ornitologia Neotropical. 012:286–296.

Vasconcelos, M., and J. Lombardi. 2001. Hummingbirds and their flowers in the campos rupestres of southern

Espinhaco range, Brazil. Melopsittacus 4:3–30.

Wolowski, M., T.-L. Ashman, and L. Freitas. 2013a. Community-wide assessment of pollen limitation in hummingbird-pollinated plants of a tropical montane rain forest. Annals of Botany. 112:903–10.

Wolowski, M., C. F. Saad, T.-L. Ashman, and L. Freitas. 2013b. Predominance of self-compatibility in hummingbird-pollinated plants in the Neotropics. Naturwissenschaften 100:69–79.
